# Gait performance of adolescent mice assessed by the CatWalk XT depends on age, strain and sex and correlates with speed and body weight

**DOI:** 10.1038/s41598-021-00625-8

**Published:** 2021-11-01

**Authors:** Claudia Pitzer, Barbara Kurpiers, Ahmed Eltokhi

**Affiliations:** 1 Interdisciplinary Neurobehavioral Core, Heidelberg University, Heidelberg, Germany; 2 Department of Pharmacology, University of Washington, Seattle, USA

**Keywords:** Neuroscience, Motor control

## Abstract

The automatization of behavioral tests assessing motor activity in rodent models is important for providing robust and reproducible results and evaluating new therapeutics. The CatWalk system is an observer-independent, automated and computerized technique for the assessment of gait performance in rodents. This method has previously been used in adult rodent models of CNS-based movement disorders such as Parkinson’s and Huntington’s diseases. As motor and gait abnormalities in neuropsychiatric disorders are observed during infancy and adolescence, it became important to validate the CatWalk XT in the gait analysis of adolescent mice and unravel factors that may cause variations in gait performance. Three adolescent wild-type inbred mouse strains, C57BL/6N, DBA/2 and FVB/N, were tested using the CatWalk XT (Version 10.6) for suitable detection settings to characterize several gait parameters at P32 and P42. The same detection settings being suitable for C57BL/6N and DBA/2 mice allowed a direct comparison between the two strains. On the other hand, due to their increased body weight and size, FVB/N mice required different detection settings. The CatWalk XT reliably measured the temporal, spatial, and interlimb coordination parameters in the investigated strains during adolescence. Additionally, significant effects of sex, development, speed and body weight within each strain confirmed the sensitivity of motor and gait functions to these factors. The CatWalk gait analysis of rodents during adolescence, taking the effect of age, strain, sex, speed and body weight into consideration, will decrease intra-laboratory discrepancies and increase the face validity of rodent models of neuropsychiatric disorders.

## Introduction

Motor and coordination dysfunctions including gait abnormalities are the main diagnostic criteria for many CNS disorders such as Parkinson’s, Huntington’s and Alzheimer’s diseases, amyotrophic lateral sclerosis, and stroke (for reviews, see^1–11^). Therefore, qualitative and quantitative behavioral experiments of motor deficits including the rotarod, grip strength, inverted screen, and beam balance rod tests are often used in rodent models based on the wide range of parallels between rodents and human locomotion. Additionally, these assays are a quantifiable measure of the effectiveness of various therapeutic strategies. However, these motor tests share the limitation of being mainly observer dependent and of detecting only gross deficits in coordination and motor function^12^. For gait analysis, observational scoring as a simple low-technology tool was widely performed 20 years ago. It used rank-order structured scales to grade the severity of rodent gait abnormalities from video recordings using visual observations^13^. Since these scales have been developed by individual laboratories, scoring systems were relatively inconsistent and observer biased, and provided only semi-quantitative gait descriptions^13^. On the other hand, several gait parameters have been studied using different experimental setups including the electric grid^14^ and footprint analysis^15–18^. In recent years, technological advances and automated systems provide observer-independent quantitative assessments of spatiotemporal patterns. Although quadrupedal gait patterns used by rodents vary significantly from bipedal human patterns, the conceptual basis for gait analysis remains analogous. Therefore, advanced technologies for rodent gait analysis are critical for better modeling human disorders.

The CatWalk is an automated and computerized gait analysis system that utilizes a restricted pathway to encourage the analysis of unidirectional movement^19^. It allows the objective quantification of multiple gait parameters simultaneously, as well as the spatial and temporal aspects of interlimb coordination^20^. Rodents traverse a glass plate that is illuminated by a green light. The green light enters at the long edge of the plate and is completely internally reflected. Only in those areas, where the animal's paws make contact with the glass plate, the light is scattered. Paws are captured by a high-speed camera placed underneath the plate, and digital images from the paws are produced by the CatWalk software. The system provides a deeper understanding of functional motor impairment by the automatic analysis of several different parameters. This method has previously been used in rodent models of spinal cord injury^20–22^, pain^23–25^, sciatic nerve injury^26,27^, traumatic brain injury^12^, and for the behavioral characterization of rodent models of CNS-based movement disorders^28–32^.

In all behavioral studies using the CatWalk system, adult rodents were used, taking advantage of the easy handling and mature motor function. Since motor and gait abnormalities in neuropsychiatric disorders such as autism spectrum and attention deficit hyperactivity disorders are well documented to appear early in life^33–38^, and neuromotor dysfunction in schizophrenia is present during adolescence^39,40^, performing gait analysis in adult rodent models of neuropsychiatric disorders may miss valuable information about the impact of synaptic maturation during development on the motor ability and learning. In our previous study, we examined the ability of adolescent mice from three wild-type inbred strains, C57BL/6N, DBA/2 and FVB/N, to perform several behavioral tests assessing motor and coordination function including the inverted screen, cliff avoidance reaction, rotarod, and voluntary wheel running tests^41^. These behavioral tests revealed strain- and sex-specific effects, highlighting the potential advantages and disadvantages of individual strains in a specific behavioral test battery. In the present study, we extended the motor characterization of the aforementioned strains by performing a complete gait analysis during adolescence using the CatWalk XT. The experiments were performed during the developmental time window that matches the onset of neuropsychiatric symptoms in human patients before or around puberty corresponding to P42 in mice^42,43^. Since behavioral results are known to be sensitive to small developmental progress during adolecense^44,45^, we compared the gait performance at two developmental stages, P32 and P42, to investigate the effect of a 10-day difference during adolescence on the gait performance of mice. We also tested both male and female mice to assess differences in gait characteristics between sexes. Additionally, we examined whether the studied gait parameters are directly correlated with average speed and body weight. Our study provides useful insights for behavioral experimental designs and presents reference data on gait performance when using young mice for preclinical investigations in neuropsychiatric indications.

## Materials and methods

### Animals and housing conditions

Animals and housing conditions were similar to our previous studies^41,45^. The experiment was conducted in strict compliance with national and international guidelines for the Care and Use of Laboratory Animals. The CatWalk XT gait analysis was approved by the animal ethic committee of the Regierungspräsidium Karlsruhe (G-102/16) and was carried out in compliance with the ARRIVE guidelines.

### Experimental design and groups

The habituation and experimental procedures of the CatWalk XT were performed during the same period of the day (from 7 am to 3 pm). Mice were brought into the behavioral room half an hour before the behavioral testing. We analyzed the behavior in 2 cohorts of group-housed mice of both sexes at P32 and P42. The numbers of male and female mice per cohort and strain are listed in Table 1. After each run, the apparatus was carefully cleaned with 75% ethanol solution wetted tissue paper. The behavioral experiment was performed in a randomized manner with a separation of experiment conduction and data analysis.

**Table 1:** Mouse cohorts and numbers of adolescent mice used in the CatWalk XT gait analysis experiments.

Cohorts	Strains	Mice (#)		Number of litters
		Male ♂	Female♀	
P32	C57BL/6N	6	9	2
	DBA/2	12	9	3
	FVB/N	11	7	3
P42	C57BL/6N	8	10	2
	DBA/2	9	7	3
	FVB/N	8	19	3

### The CatWalk gait analysis system

**Figure 1:**
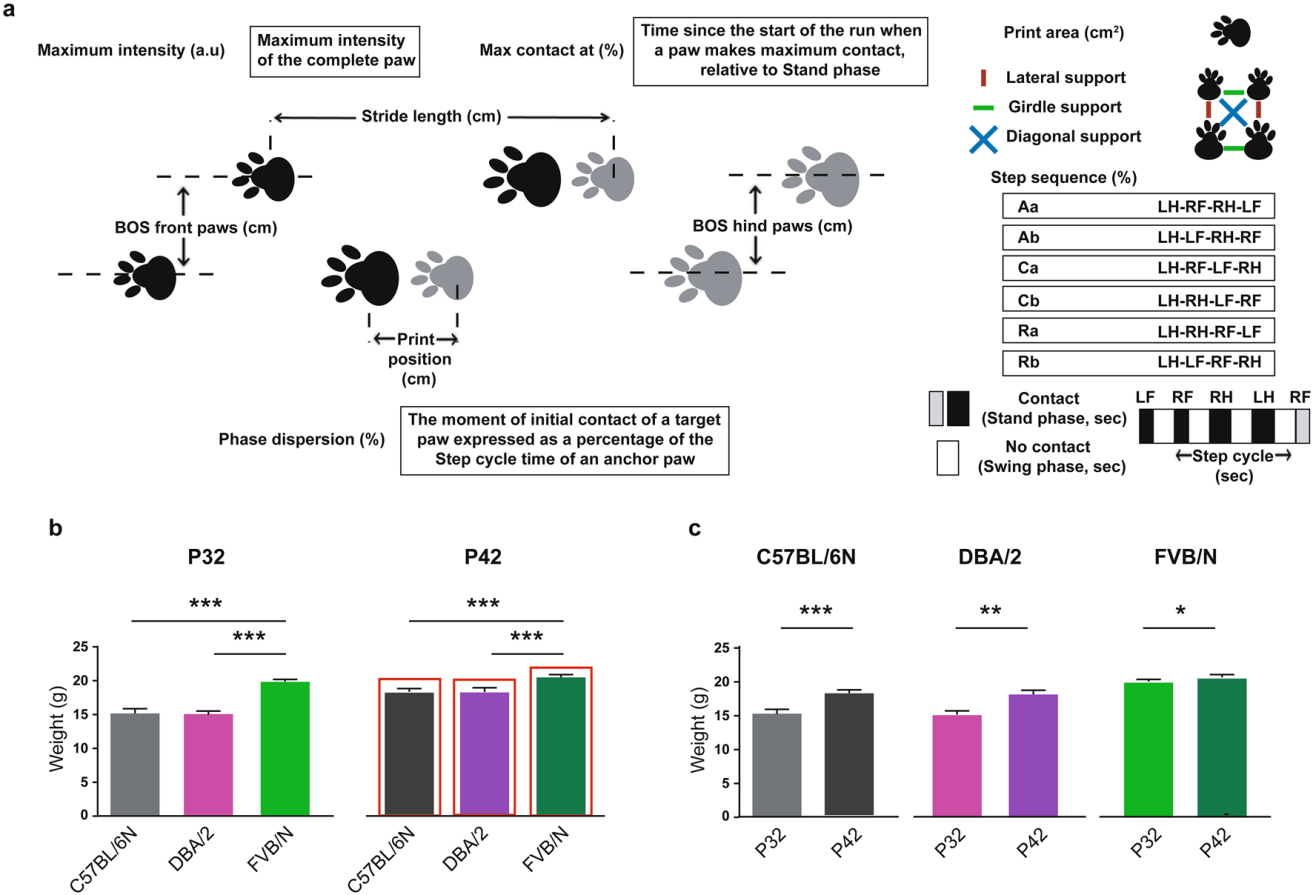
(**a**) Graphical representation of selected gait parameters. *RF* right front paw, *LF* left front paw, *RH* right hind paw, *LH* left hind paw. (**b**) FVB/N mice showed increased body weight compared to C57BL/6N and DBA/2 mice at P32 and P42. (**c**) Comparison of the body weights of mice at P32 and P42 within each strain. Two-way ANOVA followed by Tukey’s post hoc test for multiple comparisons to determine differences between the three strains and Bonferroni correction to check differences between males and females within each strain, *p ≤ 0.05, **p ≤ 0.01, ***p ≤ 0.001. A red rectangle indicates a significant difference between sexes within a strain (see Supplementary Table S1). Error bars indicate the standard error of the mean (SEM).

The CatWalk XT (version 10.6) gait analysis system (Noldus, Netherlands system) consisted of a 1.3-m black corridor on a glass plate with a green LED lit inside, and placed in a dark and silent room (< 20 lx of illumination). Using the Illuminated footprints technology, paws were captured by a high-speed video camera (100 frames per second) that was positioned underneath the glass. The experiment was performed as previously described^46^ with modifications. First, the walkway area was set according to the company’s recommendations. Mice were then habituated to the CatWalk XT and allowed to cross the corridor voluntarily for three accomplished runs at P31 and P41. To achieve an unforced type of locomotion, we did not include in our method any light stimulus, food enticement or even using the home cage as bait at the end of the walkway. On the following day, mice were tested by crossing the corridor three times. A compliant run is described as a mouse walking across the runway without stopping, turning around, or changing direction. We set up the minimum and maximum run durations to be 0.5 and 12 s, respectively. Three compliant runs made up the testing run. We have configurated different detection settings for the 3 strains. For all C57BL/6N and DBA/2 mice, the same detection settings were used (Camera Gain: 23.70, Green Intensity Threshold: 0.12, Red Ceiling Light: 17.7, and Green Walkway Light: 16.5). Due to their larger body size and weight, all FVB/N mice required other detection settings (Camera Gain: 16.99, Green Intensity Threshold: 0.10, Red Ceiling Light: 17.7, and Green Walkway Light: 16.5). For the analysis, gait parameters were automatically generated after each footprint being manually checked and respectively labeled LF (Left Front), LH (Left Hind), RF (Right Front), and RH (Right Hind) paws. For the presentation of the data, we categorized the gait parameters into 4 major groups: (a) run characteristics and kinetic parameters, (b) temporal parameters, (c) spatial parameters, (d) interlimb coordination parameters. These parameters include body and swing speeds, stand and swing times, step cycle, maximum contact at (% of stand time), print area, maximum intensity, base of support, stride length, print position, support, phase dispersion, and step sequences (for more details about the tested parameters, see Fig. 1a).

### Statistical analysis

For the comparison of the body weights of mice in Fig. 1b, two-way ANOVA was used with sex and genotype as the two factors. This was followed by Tukey’s post hoc test for multiple comparisons to determine differences between the three strains C57BL/6N, DBA/2, and FVB/N and Bonferroni correction to check differences between males and females within each strain. For the gait parameters, three compliant runs of each mouse made up the testing run. Two-way ANOVA followed by Bonferroni correction was used to compare the average values of the three runs for each C57BL/6N and DBA/2 mouse, with sex and genotype as the two factors. To compare the gait parameters at the two developmental stages, P32 and P42, within each strain, two-way ANOVA was used with sex and age as the two factors. All data were expressed as mean ± SEM. For measuring the correlation between average speed, body weight and different gait parameters, Pearson’s correlation coefficient was used separately for each strain by combining the data of the two investigated developmental stages. A *p* value ≤ 0.05 was considered statistically significant. Statistical analysis was performed using GraphPad Prism 7 and Microsoft Office Excel software. The respective numbers of male and female mice per cohort are described in Table 1.

## Results

Most of the gait parameters that we focused on in our study are shown for each paw, namely the left front (LF), left hind (LH), right front (RF), and right hind (RH) paw. The definitions and explanations for the investigated gait parameters are shown in Fig. 1a.

### Body weights of C57BL/6N, DBA/2 and FVB/N mice at P32 and P42

The body weights of mice was used as an indicator of their general condition, a guide for determining suitable detection settings, and to find its correlation to different gait parameters. The comparison between C57BL/6N, DBA/2 and FVB/N mice revealed an increase in the body weights of FVB/N mice at P32 and P42 (*p* < 0.0001) (Fig. 1b). Male C57BL/6N, DBA/2 and FVB/N mice showed significantly increased body weights compared to females at P42 (*p* < 0.0001) (Fig. 1b, Supplementary Table S1). The body weights of C57BL/6N, DBA/2 and FVB/N mice at P42 were significantly more than their respective body weights at P32 (C57BL/6N: *p* = 0.0002; DBA/2: *p* = 0.002; FVB/N: *p* = 0.014) (Fig. 1c).

### Run characteristics and spatial, temporal and interlimb coordination parameters in C57BL/6N and DBA/2 mice at P32

**Figure 2:**
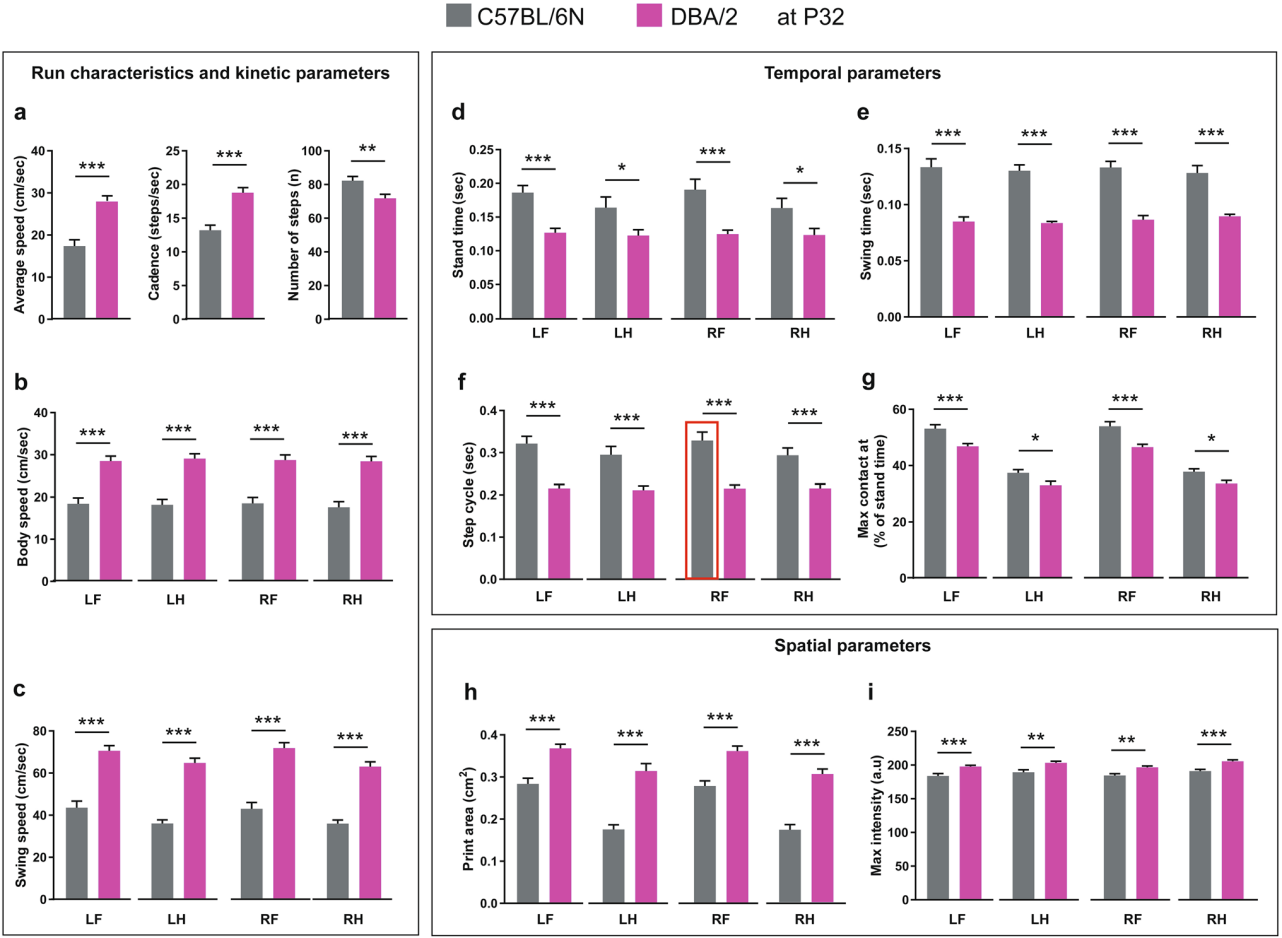
Comparison of run characteristics and kinetic, temporal and spatial parameters between C57BL/6N and DBA/2 mice at P32. (**a**) DBA/2 mice showed a significant increase in the average speed and cadence and a decreased number of steps compared to C57BL/6N mice. (**b**,**c**) DBA/2 mice showed an increase in body speed (**b**) and swing speed (**c**) in all paws compared to C57BL/6N mice (**d**–**g**) DBA/2 mice showed a significant decrease in stand time (**d**), swing time (**e**), step cycle (**f**) and maximum contact at (%) (**g**) of all paws compared to C57BL/6N mice. (**h**,**i**) DBA/2 mice showed an increase in print area (**h**) and maximum intensity (**i**) of all paws compared to C57BL/6N mice. Two-way ANOVA followed by Bonferroni post hoc test, *p ≤ 0.05, **p ≤ 0.01, ***p ≤ 0.001. A red rectangle indicates a significant difference between sexes within a strain (see Supplementary Table S1). Error bars indicate the standard error of the mean (SEM). *RF* right front, *LF* left front, *RH* right hind, *LH* left hind.

For the presentation of the data, we categorized the gait parameters into 4 major groups: (1) Run characteristics and kinetic parameters, (2) temporal parameters, (3) spatial parameters, (4) interlimb coordination parameters. The same applied detection settings for both C57BL/6N and DBA/2 mice allowed a direct comparison of their gait characteristics. DBA/2 mice showed increased average speed and cadence and a reduced number of steps compared to C57BL/6N mice at P32 (Fig. 2a). The increased average speed of DBA/2 mice was extended to an increase in the body and swing speeds of all paws compared to C57BL/6N mice (Fig. 2b,c). Moreover, DBA/2 mice showed less stand time, swing time and step cycle of all paws than C57BL/6N mice (Fig. 2d–f). DBA/2 mice also showed a reduced time for all paws to make the maximum contact to the glass plate since the start of the run, relative to their stand time (Fig. 2g). For spatial parameters, DBA/2 revealed increased print areas and maximum intensity of all paws compared to C57BL/6N mice (Fig. 2h,i).

**Figure 3:**
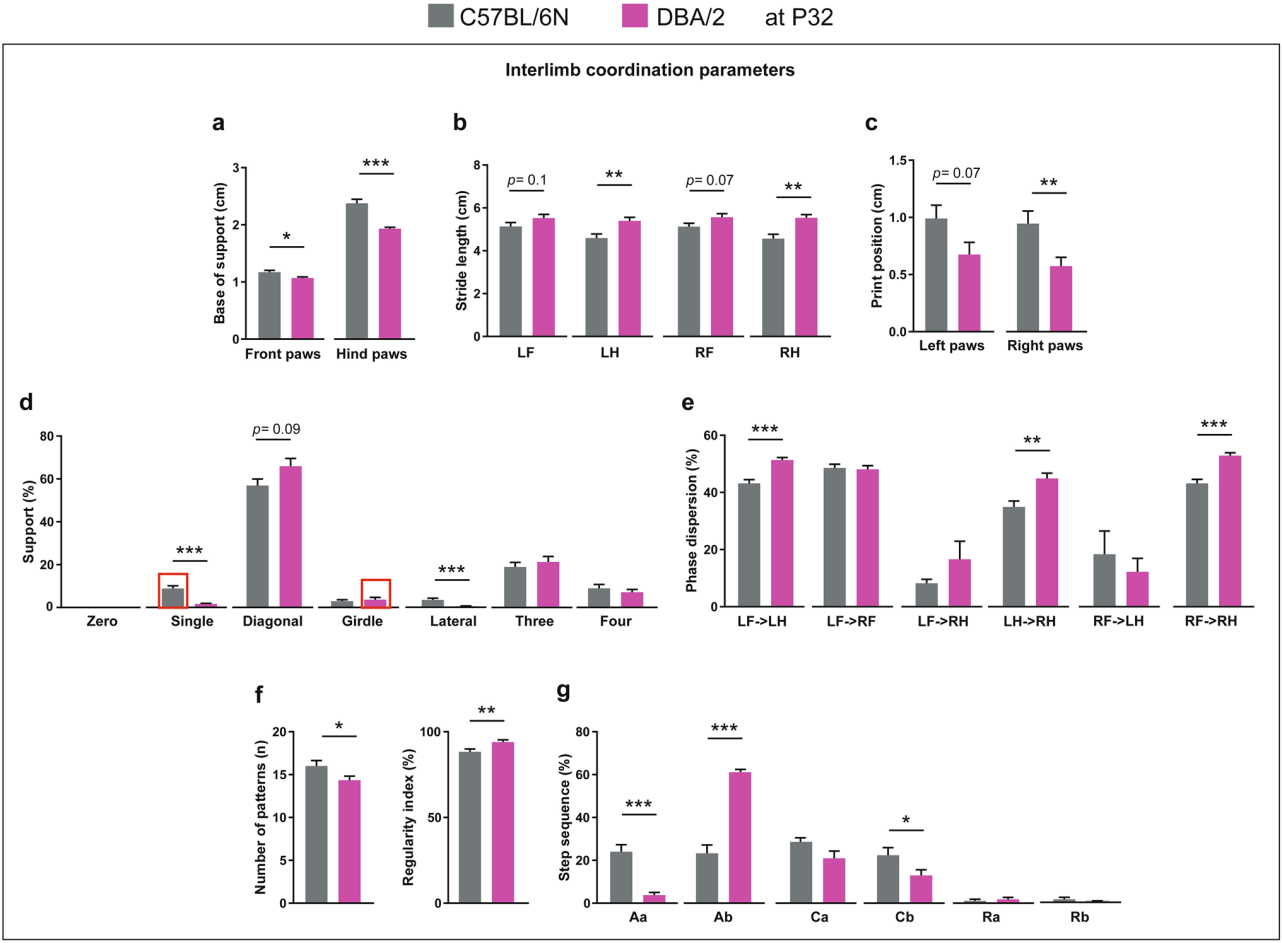
Comparison of interlimb coordination parameters between C57BL/6N and DBA/2 at P32. (**a**) DBA/2 mice showed a decrease in the base of support of both front and hind paws compared to C57BL/6N mice. (**b**) The stride lengths of LH and RH paws were more in DBA/2 than C57BL/6N mice. (**c**) The print position of the right paws was less in DBA/2 compared to C57BL/6N mice. (**d**) DBA/2 mice used less single and lateral support compared to C57BL/6N mice. (**e**) The phase dispersion percentages of LF- > LH, LH- > RH and RF- > RH were more in DBA/2 than C57BL/6N mice. (**f**) The number of patterns in DBA/2 mice was fewer, but the regularity index was higher than in C57BL/6N mice. (**g**) DBA/2 mice showed lower step sequence percentages of Aa and Cb and a higher percentage of Ab compared to C57BL/6N mice. Two-way ANOVA followed by Bonferroni post hoc test, *p ≤ 0.05, **p ≤ 0.01, ***p ≤ 0.001. A red rectangle indicates a significant difference between sexes within a strain (see Supplementary Table S1). Error bars indicate the standard error of the mean (SEM). *RF* right front, *LF* left front, *RH* right hind, *LH* left hind.

Comparing interlimb coordination parameters between C57BL/6N and DBA/2 mice at P32 revealed a lower base of support of front and hind paws of DBA/2 mice (Fig. 3a). For stride length, only the hind paws of DBA/2 mice showed a significant increase of stride length compared to C57BL/6N mice, with their front paws showing only a tendency towards increase (Fig. 3b). DBA/2 mice showed a significantly reduced print position of right paws and a borderline significant reduction in left paws (Fig. 3c). Both C57BL/6N and DBA/2 mice were mainly supported by two paws in a diagonal shape during the run with an increased tendency in DBA/2 mice (Fig. 3d). In contrast, C57BL6N mice revealed an increased percentage of support by one and two paws (lateral) compared to DBA/2 mice (Fig. 3d). For the phase dispersion, the moment of initial contact of a target paw expressed as a percentage of the step cycle time of an anchor paw, DBA/2 mice showed an increased percentage of phase dispersion of LF with LH as the anchor paw, LH with RH as the anchor paw, and RF with RH as the anchor paw compared to C57BL/6N mice (Fig. 3e). DBA/2 mice showed fewer numbers of patterns and increased regularity index during the run compared to C57BL/6N mice (Fig. 3f). C57BL/6N mice used a similar ratio of alternate and cruciate patterns during the run (Fig. 3g). In contrast, DBA/2 mice used mainly the alternate pattern (Ab) during the run, with significantly reduced Aa and Cb patterns compared to C57BL/6N mice (Fig. 3g).

Comparing male and female mice within the aforementioned strains revealed no difference in run characteristic, kinetic or spatial parameters. For temporal parameters, male C57BL/6N mice showed a longer step cycle of the RF paw than females (*p* = 0.046) (Supplementary Table S1). For interlimb coordination parameters, only the support percentage showed sex-biased results, with female C57BL6N mice showing more support percentage on single paw than males (*p* = 0.041) and female DBA/2 mice showing more support percentage on two paws (girdle) than males (*p* = 0.037) (Supplementary Table S1).

### Run characteristics and spatial, temporal and interlimb coordination parameters in C57BL/6N and DBA/2 mice at P42

**Figure 4:**
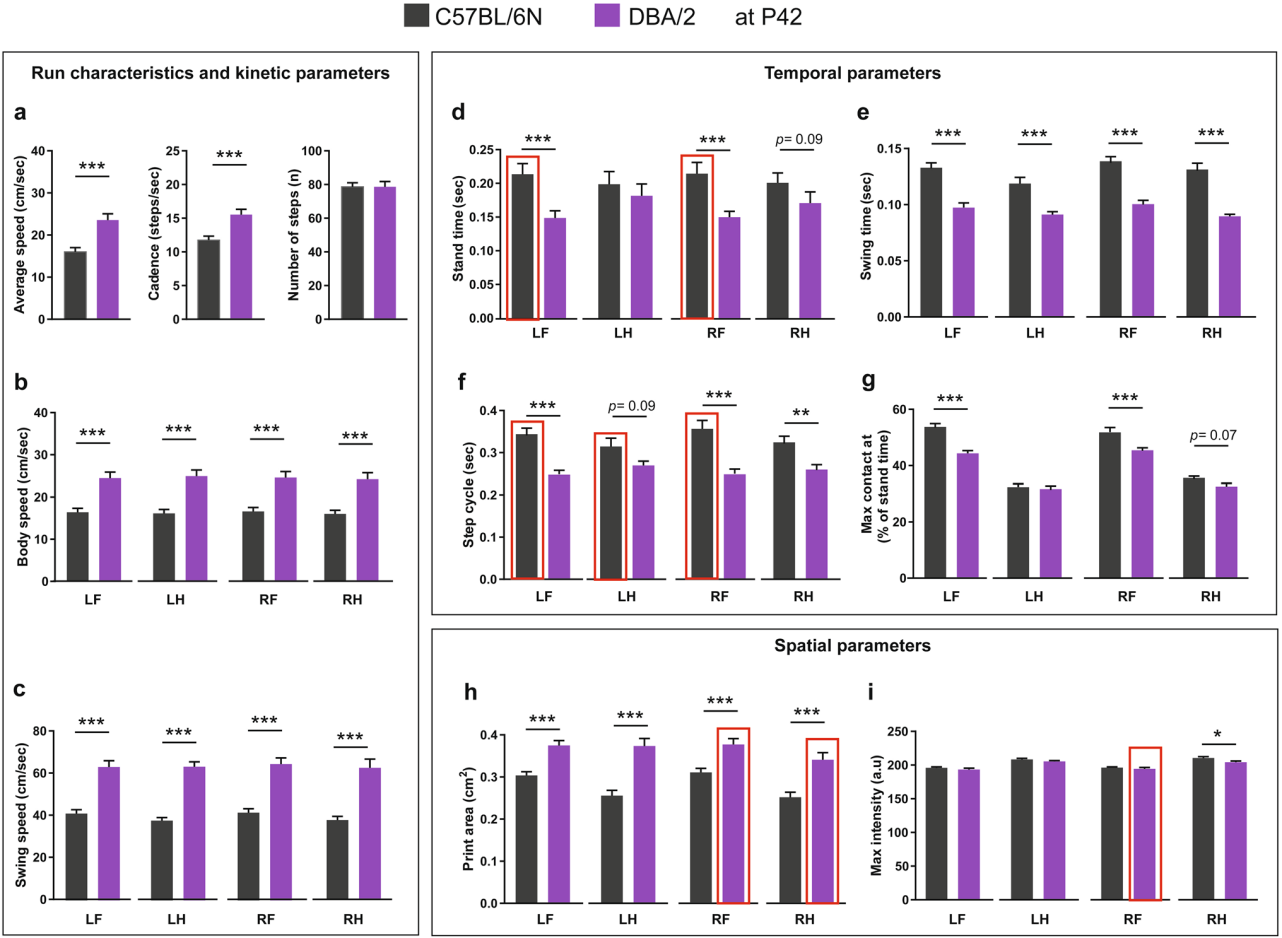
Comparison of run characteristics and kinetic, temporal and spatial parameters between C57BL/6N and DBA/2 mice at P42. (**a**) DBA/2 mice showed a significant increase in the average speed and cadence, but no difference in the number of steps compared to C57BL/6N mice. (**b**,**c**) DBA/2 mice showed an increase in the body speed (**b**) and swing speed (**c**) of all paws compared to C57BL/6N mice. (**d**) DBA/2 mice showed a significant decrease in the stand time of LF and RF paws. (**e**) DBA/2 mice showed a significant decrease in the swing time of all paws. (**f**) DBA/2 mice showed a significant decrease in the step cycle of LF, RF and RH paws. (**g**) DBA/2 mice showed a significant decrease in the maximum contact at (%) of both LF and RF paws compared to C57BL/6N mice. (**h**,**i**) DBA/2 mice showed an increase in the print area of all paws (**h**) and a decrease in the maximum intensity of the RH paw compared to C57BL/6N mice. (**i**). Two-way ANOVA followed by Bonferroni post hoc test, *p ≤ 0.05, **p ≤ 0.01, ***p ≤ 0.001. A red rectangle indicates a significant difference between sexes within a strain (see Supplementary Table S1). Error bars indicate the standard error of the mean (SEM). *RF* right front, *LF* left front, *RH* right hind, *LH* left hind.

At P42, DBA/2 mice showed increased average speed and cadence compared to C57BL/6N mice, with no difference in the number of steps (Fig. 4a). The increased average speed of DBA/2 mice was extended to an increase in the body and swing speeds of all paws compared to C57BL/6N mice (Fig. 4b,c). For the temporal parameters, the stand times of the front paws, LF and RF, of DBA/2 mice were significantly less compared to C57BL/6N mice (Fig. 4d). Additionally, the swing time and step cycle of all paws of DBA/2 mice were less compared to C57BL/6N mice (Fig. 4e,f). DBA/2 mice also showed a reduced time for the front paws, LF and RF, to make the maximum contact to the glass plate since the start of the run, relative to their stand time (Fig. 4g). For spatial parameters, DBA/2 mice had increased print areas of all paws compared to C57BL/6N mice, with no change in the maximum intensity except for the reduced maximum intensity of the RH paw (Fig. 4h,i).

**Figure 5:**
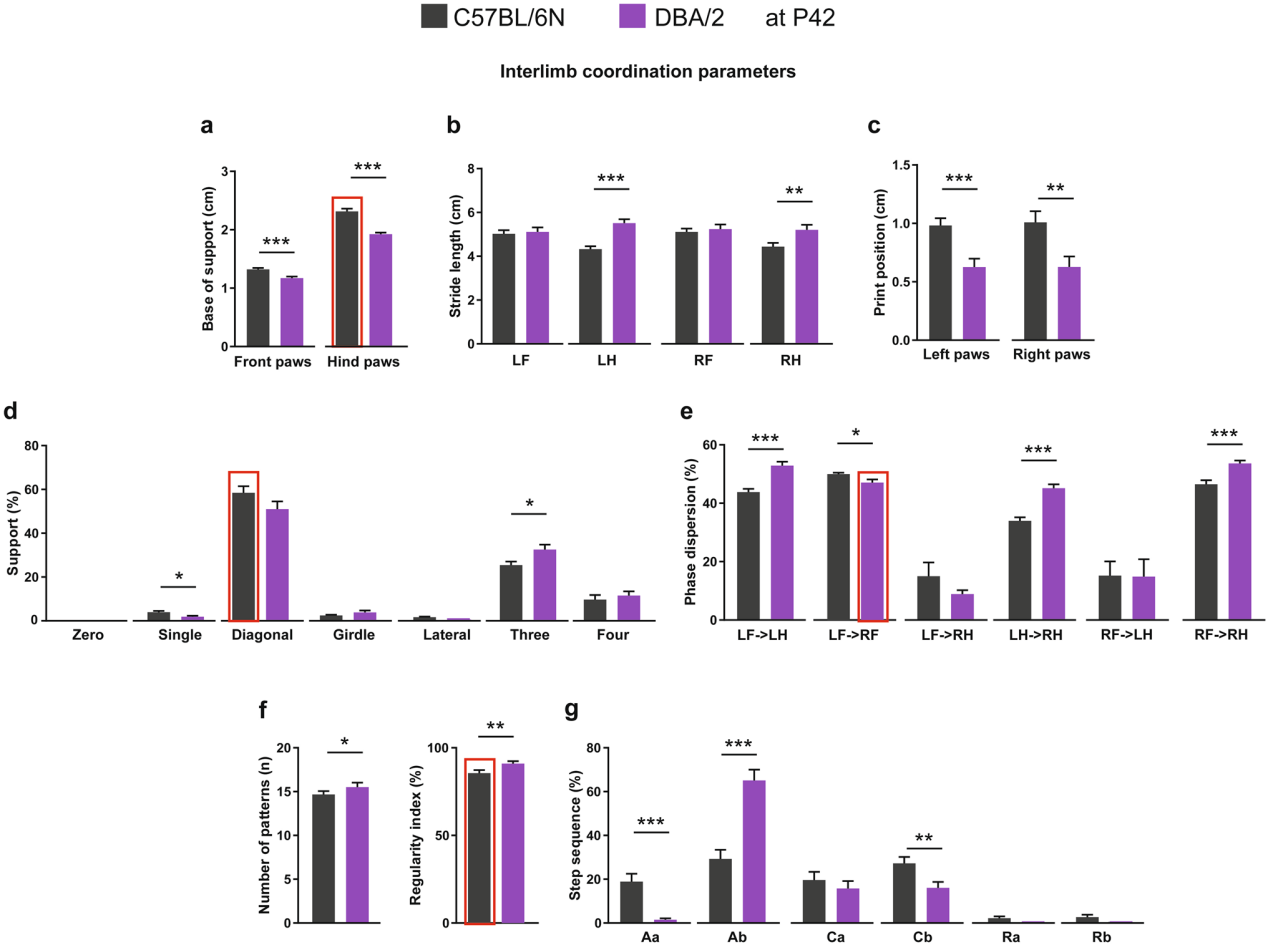
Comparison of interlimb coordination parameters between C57BL/6N and DBA/2 mice at P42. (**a**) DBA/2 mice showed a decrease in the base of support of both front and hind paws compared to C57BL/6N mice. (**b**) The stride lengths of both LH and RH paws were higher in DBA/2 than C57BL/6N mice. (**c**) The print positions of both left and right paws were lower in DBA/2 compared to C57BL/6N mice. (**d**) DBA/2 mice used less single but more three-paws support compared to C57BL/6N mice. (**e**) The phase dispersion percentages of LF- > LH, LH- > RH and RF- > RH were higher in DBA/2 than C57BL6N mice. (**f**) The number of patterns and regularity index were higher in DBA/2 than C57BL/6N mice. (**g**) DBA/2 mice showed lower step sequence percentages of Aa and Cb and a higher percentage of Ab compared to C57BL/6N mice. Two-way ANOVA followed by Bonferroni post hoc test, *p ≤ 0.05, **p ≤ 0.01, ***p ≤ 0.001. A red rectangle indicates a significant difference between sexes within a strain (see Supplementary Table S1). Error bars indicate the standard error of the mean (SEM). *RF* right front, *LF* left front, *RH* right hind, *LH* left hind.

Comparing interlimb coordination parameters between C57BL/6N and DBA/2 strains at P42 unraveled the lower base of support for front and hind paws of DBA/2 mice (Fig. 5a). For stride length, only the hind paws of DBA/2 mice showed a significant increase compared to C57BL/6N mice (Fig. 5b). DBA/2 mice showed significantly reduced print positions of both right and left paws (Fig. 5c). Both C57BL/6N and DBA/2 mice were mainly supported by two paws in a diagonal shape during the run (Fig. 5d). C57BL6N mice showed an increased percentage of support by one and decreased support by three paws during the run compared to DBA/2 mice (Fig. 5d). For the phase dispersion, DBA/2 mice showed an increased percentage of phase dispersion of LF with LH as the anchor paw, LH with RH as the anchor paw, RF with RH as the anchor paw, and reduced phase dispersion of LF with RF as the anchor paw compared to C57BL/6N mice (Fig. 5e). DBA/2 mice showed increased numbers of patterns and regularity index during the run (Fig. 5f). Similar to P32 C57BL/6N mice, P42 C57BL/6N mice used a similar ratio of alternate and cruciate patterns during the run (Fig. 5g). In contrast, DBA/2 mice used mainly the alternate pattern (Ab) during the run with significantly reduced Aa and Cb patterns compared to C57BL/6N mice (Fig. 5g).

Comparing male and female mice within the aforementioned strains revealed no difference in the run characteristic or kinetic parameters. On the other hand, for temporal parameters, the stand time and step cycle of mainly front paws of males C57BL/6N mice was more than of females (Stand time: LF: *p* = 0.004, RF: *p* = 0.005; Step cycle: LF: *p* = 0.007, LH: *p* = 0.03, RF: *p* = 0.008) (Supplementary Table S1). For spatial parameters, the right paws of DBA/2 male mice showed increased print areas compared to females (RF: *p* = 0.028, RH: *p* = 0.003), with only the RF paw of male DBA/2 mice showing an increase in the maximum intensity (*p* = 0.005) (Supplementary Table S1). For interlimb coordination parameters, the base of support of hind paws in male C57BL6N mice was more than females (*p* < 0.0001). The support percentage showed sex-biased results with females C57BL/6N mice showing more support on two paws (diagonal) than males (*p* = 0.049). For the regularity index, female C57BL/6N mice showed an increased regularity index than males (*p* = 0.008). For DBA/2 mice, only the phase dispersion of LF with RF as the anchor paw showed a sex difference with females showing a higher percentage than males (*p* = 0.022) (Supplementary Table S1).

### Comparison of running characteristics and spatial, temporal and interlimb coordination parameters in C57BL/6N mice between P32 and P42

The comparison between P32 and P42 C57BL/6N mice revealed no difference in the run characteristics or kinetic parameters (Supplementary Figure S1a–c), and for the temporal parameters, no difference in stand time, swing time or step cycle was found between the two ages (Supplementary Figure S1d–f). On the other hand, only the LH paw of P42 C57BL/6N mice showed a decreased time to make the maximum contact to the glass plate since the start of the run, relative to their stand time (Supplementary Figure S1g). For the spatial parameters, P42 mice showed increased print areas of the hind paws and increased maximum intensity of all paws compared to P32 mice (Supplementary Figure S1h,i).

Comparing interlimb coordination parameters between P32 and P42 C57BL/6N mice revealed an increased base of support of the front paws in P42 mice (Supplementary Figure S2a). No difference was found between the two ages in the stride length, print position, phase dispersion, step sequence or number of patterns (Supplementary Figure S2b,c,e–g). On the other hand, P42 C57BL/6N mice showed less support on one and two paws (lateral) and increased support on three paws compared to P32 (Supplementary Figure S2d).

### Comparison of running characteristics and spatial, temporal and interlimb coordination parameters in DBA/2 mice between P32 and P42

The comparison between P32 and P42 DBA/2 mice revealed a decrease in the average speed and cadence at P42, which was extended to a tendency of decreased body speed of all paws and decreased swing speed of only front paws (Supplementary Figure S3a–c). For the temporal parameters, the stand and swing times and step cycle of most paws, in principle, were longer at P42 than P32, but no difference was found regarding the time required to make the maximum contact to the glass plate (Supplementary Figure S3d–g). For the spatial parameters, no difference in the print area or maximum intensity of all paws was found between the two ages except a significant increase in the print area of the LH paw at P42 (Supplementary Figure S3h–i).

Comparing interlimb coordination parameters between P32 and P42 DBA/2 mice revealed an increased base of support of front paws in P42 mice (Supplementary Figure S4a). No difference was found between the two ages in the stride length, print position, phase dispersion, step sequence or number of patterns (Supplementary Figure S4b,c,e–g). On the other hand, P42 DBA/2 mice showed less support on two paws (diagonal and lateral) and increased support on three paws compared to P32 mice (Supplementary Figure S4d).

### Run characteristics and spatial, temporal and interlimb coordination parameters in FVB/N mice at P32 and P42

**Figure 6:**
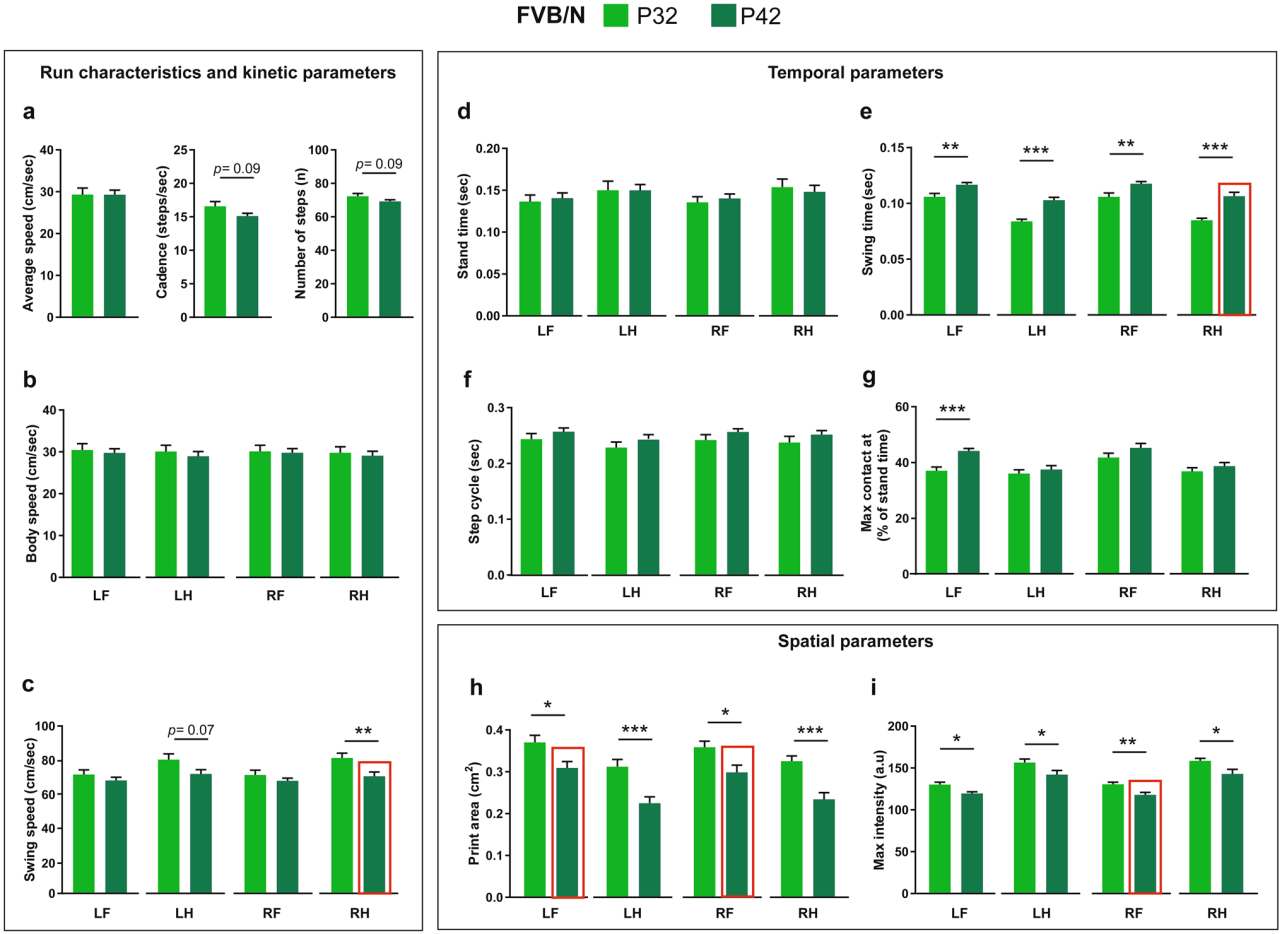
Comparison of run characteristics and kinetic, temporal and spatial parameters between P32 and P42 FVB/N mice. (**a**,**b**) No differences were seen in the average speed, cadence, number of steps (**a**) or body speed (**b**) between P32 and P42 FVB/N mice. (**c**) The swing speed of the RH paw was significantly less at P42 than P32. (**d**) No difference was seen in the stand time between P32 and P42 FVB/N mice. (**e**) The swing time of all paws at P42 was longer than at P32. (**f**) No difference was seen in the step cycle between P32 and P42 FVB/N mice. (**g**) The maximum contact at (%) of the LF paw was significantly higher at P42 than at P32. (**h**,**i**) The print area (**h**) and the maximum intensity (**i**) at P42 were lower than at P32. Two-way ANOVA followed by Bonferroni post hoc test, *p ≤ 0.05, **p ≤ 0.01, ***p ≤ 0.001. A red rectangle indicates a significant difference between sexes within a strain (see Supplementary Table S1). Error bars indicate the standard error of the mean (SEM). *RF* right front, *LF* left front, *RH* right hind, *LH* left hind.

The comparison in the run characteristics of FVB/N mice between P32 and P42 revealed no difference in the average speed, but a tendency of a reduction of cadence and number of steps at P42 (Fig. 6a). For the body speed, no difference was revealed in all paws between the two ages (Fig. 6b). On the other hand, there was a reduction of swing speed in hind paws, which reached significance only for the RH paw (Fig. 6c). For the temporal parameters, the stand time and step cycle did not show any difference between the two ages (Fig. 6d,f). In contrast, P42 FVB/N mice showed an increased swing time of all paws compared to P32 ones (Fig. 6e). Only the LF paw in P42 mice showed an increase in the time required to make the maximum contact to the glass plate since the start of the run, relative to the stand time (Fig. 6g). For the spatial parameters, P42 FVB/N mice showed lower print areas and maximum intensities of all paws than P32 mice (Fig. 6h,i).

**Figure 7:**
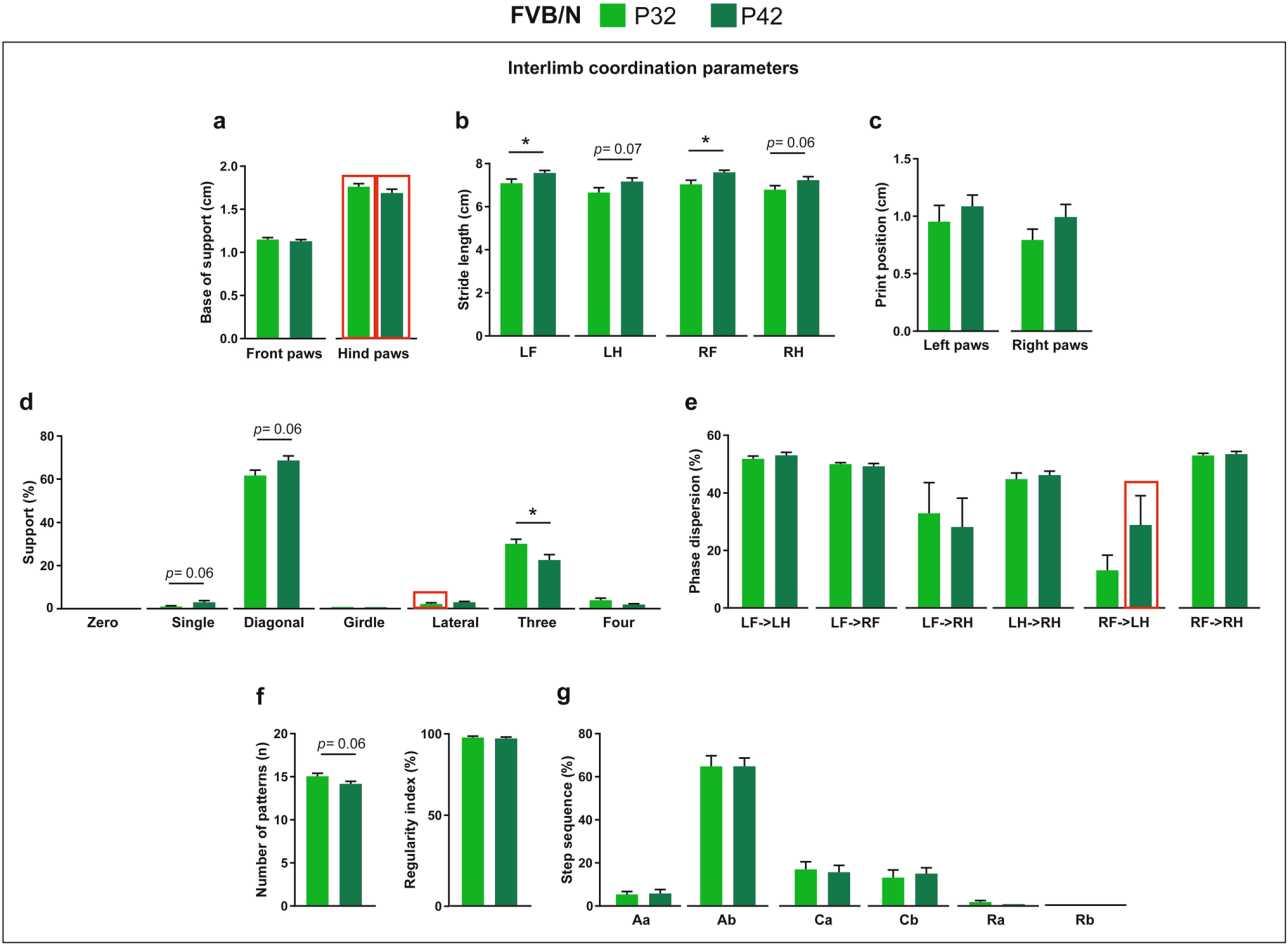
Comparison of interlimb coordination parameters between P32 and P42 FVB/N mice. (**a**) No difference was seen in the base of support of either front or hind paws between P32 and P42. (**b**) The stride lengths of the front paws at P42 were significantly longer than at P32. The stride lengths of hind paws at P42 showed only a borderline significant increase compared to P32. (**c**) No difference was seen in the print position of either left or right paws between P32 and P42. (**d**) The percentage of support on three paws was significantly lower at P42 than at P32. The support on single and diagonal was borderline significantly increased at P42. (**e–g**) No differences were seen in the phase dispersion (**e**), number of patterns, regularity index (**f**) or step sequence (**g**) between P32 and P42 FVB/N mice. Two-way ANOVA followed by Bonferroni post hoc test, *p ≤ 0.05. A red rectangle indicates a significant difference between sexes within a strain (see Supplementary Table S1). Error bars indicate the standard error of the mean (SEM). *RF* right front, *LF* left front, *RH* right hind, *LH* left hind.

Comparing interlimb coordination parameters between P32 and P42 FVB/N mice revealed no difference in the base of support or print position of paws (Fig. 7a,c). In contrast, the stride length of the front paws of P42 FVB/N mice was significantly higher than that of P32 mice, with hind paws showing only a tendency of increase (Fig. 7b). For the phase dispersion, regularity index and step sequence percentage, no difference was found between the two ages (Fig. 7e–g). Comparing the support of mice on paws, P42 mice showed a tendency of increased single and diagonal support and a significantly decreased support on three paws (Fig. 7d).

Comparing male and female FVB/N mice revealed no difference in the run characteristic or kinetic parameters except for the increased swing speed in the RH paw of P42 male FVB/N mice (*p* = 0.032) (Supplementary Table S1). For the spatial parameters, the front paws of P42 male mice showed an increased print area compared to females (LF: *p* = 0.034; RF: *p* = 0.029) and increased maximum intensity of the RF paw (*p* = 0.047). For interlimb coordination parameters, the base of support of the hind paws at P32 and P42 showed sex difference with a higher base of support in male than female FVB/N mice (P32: *p* = 0.003; P42: *p* < 0.0001) (Supplementary Table S1).

### Correlation between the speed and gait parameters

As suggested by several studies, the speed of adult mice affects gait parameters^19,46–50^. To test this effect in adolescent mice, we combined the gait parameter data of P32 and P42 for each strain and calculated Pearson's correlation to the average speed of mice during runs. The correlation between speed and gait parameters was very similar in the three investigated strains albeit with some little differences, which highlights the speed of the mouse as a strong predictor of its gait performance. The cadence, body and swing speeds, stride lengths of all paws, support on diagonal paws and regularity index showed a positive correlation to the speed of mice (Table 2). In contrast, the number of steps, stand times of all paws, swing times of front paws, step cycles of all paws, support on three and four paws and the number of sequence patterns showed a negative correlation to the speed of mice during runs (Table 2). While the speed of DBA/2 mice showed a negative correlation with print positions of both left and right paws, the speed of C57BL/6N mice showed a significant correlation to print position of only right paws, and the speed of FVB/N mice showed a significant correlation to print position of only left paws. Moreover, the speed of C57BL/6N mice was characterized by additional correlations with maximum contact at (% of stand time) and maximum intensity of LH and phase dispersions of LF- > LH and LH- > RH, suggesting a specific strong correlation between speed and the left hind paw of C57BL/6N mice (Table 2).

**Table 2:** Correlation between the speed and gait parameters.

Parameters	C57BL/6N		DBA/2		FVB/N	
	R score	*P* value	R score	*P* value	R score	*P* value
Cadence	0.8461	**< 0.00001**	0.8796	**< 0.00001**	0.8516	**< 0.00001**
Number of steps	− 0.8632	**< 0.00001**	− 0.8643	**< 0.00001**	− 0.7660	**< 0.00001**
Body Speed LF	0.9832	**< 0.00001**	0.9641	**< 0.00001**	0.9734	**< 0.00001**
Body Speed LH	0.9550	**< 0.00001**	0.9621	**< 0.00001**	0.9629	**< 0.00001**
Body Speed RF	0.9744	**< 0.00001**	0.9635	**< 0.00001**	0.9725	**< 0.00001**
Body Speed RH	0.9674	**< 0.00001**	0.9692	**< 0.00001**	0.9695	**< 0.00001**
Swing speed LF	0.9311	**< 0.00001**	0.8467	**< 0.00001**	0.8534	**< 0.00001**
Swing speed LH	0.6435	**0.0001**	0.6680	**< 0.00001**	0.6659	**0.00001**
Swing speed RF	0.9258	**< 0.00001**	0.9054	**< 0.00001**	0.8323	**< 0.00001**
Swing speed RH	0.6939	**< 0.00001**	0.6291	**0.0000**	0.6749	**< 0.00001**
Stand time LF	− 0.7727	**< 0.00001**	− 0.8030	**< 0.00001**	− 0.8172	**< 0.00001**
Stand time LH	− 0.8080	**< 0.00001**	− 0.8285	**< 0.00001**	− 0.8309	**< 0.00001**
Stand time RF	− 0.8039	**< 0.00001**	− 0.8523	**< 0.00001**	− 0.8485	**< 0.00001**
Stand time RH	− 0.7537	**< 0.00001**	− 0.7975	**< 0.00001**	− 0.8148	**< 0.00001**
Swing time LF	− 0.5926	**0.0003**	− 0.4047	**0.0131**	− 0.6271	**0.0001**
Swing time LH	− 0.1678	0.3529	− 0.2840	0.0885	− 0.2318	0.1818
Swing time RF	− 0.7248	**< 0.00001**	− 0.4307	**0.0079**	− 0.6690	**0.00001**
Swing time RH	− 0.0522	0.7738	− 0.1161	0.4942	− 0.2489	0.1509
Step cycle LF	− 0.8604	**< 0.00001**	− 0.8205	**< 0.00001**	− 0.8699	**< 0.00001**
Step cycle LH	− 0.7533	**< 0.00001**	− 0.8106	**< 0.00001**	− 0.7893	**< 0.00001**
Step cycle RF	− 0.8762	**< 0.00001**	− 0.8166	**< 0.00001**	− 0.8391	**< 0.00001**
Step cycle RH	− 0.7470	**< 0.00001**	− 0.8274	**< 0.00001**	− 0.8235	**< 0.00001**
Max contact at LF	− 0.2629	0.1408	0.2233	0.1840	− 0.0884	0.6152
Max contact at LH	0.3623	**0.0383**	− 0.0521	0.7599	0.0945	0.5892
Max contact at RF	0.1898	0.2901	0.3510	**0.0332**	0.1332	0.4456
Max contact at RH	− 0.2387	0.1823	0.2326	0.1659	0.1256	0.4722
Print area LF	0.1671	0.3527	0.0546	0.7482	0.0606	0.7295
Print area LH	− 0.3533	**0.0439**	− 0.5743	**0.0002**	− 0.0588	0.7407
Print area RF	− 0.0255	0.8902	0.1069	0.5289	− 0.0151	0.9318
Print area RH	− 0.3047	0.0854	− 0.2798	0.0945	− 0.2112	0.2237
Maximum intensity LF	0.1334	0.4592	0.2686	0.1080	− 0.0483	0.7842
Maximum Intensity LH	− 0.3596	**0.0402**	− 0.0392	0.8187	− 0.0299	0.8687
Maximum intensity RF	− 0.1100	0.5423	0.1112	0.5123	− 0.0126	0.9455
Maximum intensity RH	− 0.0421	0.8165	− 0.0601	0.7243	− 0.1510	0.3866
Base of support Front	0.0184	0.9190	− 0.4319	**0.0077**	− 0.3350	**0.0492**
Base of support hind	− 0.1330	0.4606	0.0689	0.6853	− 0.2185	0.2084
Stride length LF	0.8637	**< 0.00001**	0.8012	**< 0.00001**	0.6668	**0.00001**
Stride length LH	0.7763	**< 0.00001**	0.7089	**< 0.00001**	0.6217	**0.0001**
Stride length RF	0.8879	**< 0.00001**	0.8042	**< 0.00001**	0.6415	**0.00001**
Stride length RH	0.7829	**< 0.00001**	0.8576	**< 0.00001**	0.6720	**< 0.00001**
Print position left	− 0.3255	0.0650	− 0.3423	**0.0383**	− 0.3606	**0.0337**
Print position right	− 0.4614	**0.0069**	− 0.5312	**0.0007**	− 0.2749	0.1112
Support zero	0.0325	0.8575	0.0167	0.9219	0.3238	0.0578
Support single	0.6222	**0.0001**	0.2028	0.2287	0.1294	0.4588
Support diagonal	0.6831	**0.00001**	0.8550	**< 0.00001**	0.6845	**< 0.00001**
Support girdle	− 0.0964	0.5951	0.0023	0.9892	− 0.2282	0.1877
Support lateral	0.0222	0.9024	− 0.0550	0.7465	0.2800	0.1033
Support three	− 0.7791	**< 0.00001**	− 0.8736	**< 0.00001**	− 0.5580	**0.0005**
Support four	− 0.6018	**0.0002**	− 0.6542	**0.00001**	− 0.6103	**0.0001**
Phase dispersion LF-LH	0.3799	**0.0292**	0.1663	0.3253	0.0966	0.5809
Phase dispersion LF-RF	0.1547	0.3900	0.2410	0.1507	− 0.0422	0.8107
Phase dispersion LF-RH	− 0.1538	0.3953	− 0.0824	0.6295	− 0.0492	0.7798
Phase dispersion LH-RH	0.4273	**0.0131**	0.2471	0.1404	0.0391	0.8235
Phase dispersion RF-LH	0.1257	0.4858	− 0.2308	0.1709	− 0.1576	0.3678
Phase dispersion RF-RH	0.3328	0.0584	0.1833	0.2775	− 0.1372	0.4326
Number of patterns	− 0.5750	**0.0005**	− 0.8048	**< 0.00001**	− 0.6559	**0.00001**
Regularity index	0.5062	**0.0027**	0.5884	**0.0001**	0.3758	**0.0261**
Step sequence-Aa	0.0866	0.6318	0.3506	**0.0334**	0.0399	0.8200
Step sequence-Ab	− 0.1858	0.3027	− 0.2114	0.2100	− 0.1402	0.4225
Step sequence-Ca	0.3785	**0.0299**	0.0594	0.7269	− 0.0664	0.7064
Step sequence-Cb	− 0.2816	0.1132	0.1345	0.4274	0.2688	0.1184
Step sequence-Ra	− 0.1775	0.3244	− 0.0305	0.8601	− 0.1313	0.4532
Step sequence-Rb	− 0.0533	0.7696	− 0.2242	0.1826	NA	NA

The correlation was measured using Pearson’s correlation coefficient for each strain. The bold values indicate significant results.

### Correlation between body weight and gait parameters

To test whether the body weights of mice can be a factor in determining the gait characteristics, we added the gait parameter data at P32 and P42 together for each strain and calculated the Pearson's correlation to the body weights of mice. For C57BL6N and DBA/2 mice, the print areas and maximum intensities of all paws showed a positive correlation with the body weights of mice (Table 3). Additionally, in C57BL/6N strain, the body weight showed a positive correlation with the base of support and print position of paws and a negative correlation with the number of patterns and regularity index (Table 3). Notably, in C57BL/6N strain, the support on single and lateral paws had a negative correlation with the body weights of mice in contrast to the support on three paws that showed a positive correlation. On the other hand, in DBA/2 mice, only the support on a single paw and four paws showed negative and positive correlations with the body weight, respectively. For the FVB/N strain, only the base of support on the hind paw and the phase dispersion of LH- > RH had a positive correlation with the body weight. In contrast, the phase dispersion of RF- > LH and Ra pattern had a negative correlation (Table 3).

**Table 3:** Correlation between the body weight and gait parameters.

Parameters	C57BL/6N		DBA/2		FVB/N	
	R score	*P* value	R score	*P* value	R score	*P* value
Average speed	− 0.1553	0.3891	− 0.2478	0.1405	0.1473	0.3984
Cadence	− 0.2198	0.2208	− 0.3207	0.0535	0.0418	0.8116
Number of steps	− 0.1146	0.5276	0.1345	0.4274	− 0.2879	0.0946
Body Speed LF	− 0.1854	0.3027	− 0.1856	0.2730	0.1593	0.3607
Body Speed LH	− 0.1929	0.2844	− 0.1662	0.3261	0.1342	0.4421
Body Speed RF	− 0.1851	0.3027	− 0.2115	0.2100	0.1581	0.3644
Body Speed RH	− 0.1815	0.3134	− 0.1792	0.2891	0.1293	0.4591
Swing speed LF	− 0.0433	0.8122	− 0.1119	0.5131	0.2299	0.1840
Swing speed LH	0.2658	0.1349	0.1511	0.3720	0.3086	0.07128
Swing speed RF	− 0.1064	0.5571	− 0.1188	0.4867	0.1155	0.5088
Swing speed RH	0.0897	0.6196	0.1580	0.3503	0.2423	0.1608
Stand time LF	0.2867	0.1057	0.1954	0.2465	0.0906	0.6047
Stand time LH	0.2684	0.1310	0.2658	0.1118	0.0778	0.6569
Stand time RF	0.1669	0.3532	0.3169	0.0560	− 0.0281	0.8731
Stand time RH	0.2259	0.2062	0.2620	0.1172	0.0769	0.6606
Swing time LF	− 0.0112	0.9516	0.2853	0.0870	− 0.1903	0.2743
Swing time LH	− 0.3279	0.0632	− 0.0708	0.6806	− 0.1679	0.3376
Swing time RF	0.2473	0.1653	0.0921	0.5877	− 0.0276	0.8776
Swing time RH	− 0.0608	0.7401	− 0.0966	0.5719	− 0.1270	0.4672
Step Cycle LF	0.2020	0.2596	0.2734	0.1016	0.0335	0.8485
Step Cycle LH	0.1310	0.4674	0.2542	0.1290	0.1021	0.5595
Step Cycle RF	0.2011	0.2618	0.2641	0.1142	− 0.0432	0.8063
Step Cycle RH	0.1231	0.4949	0.292	0.0795	0.0680	0.6979
Max Contact at (%) LF	0.1273	0.4802	− 0.3509	**0.0337**	− 0.0598	0.7364
Max Contact at (%) LH	− 0.3320	0.0591	− 0.1562	0.3565	− 0.0289	0.8731
Max Contact at (%) RF	− 0.3258	0.0650	0.0094	0.9560	− 0.1059	0.5483
Max Contact at (%) RH	− 0.1497	0.4079	− 0.1685	0.3203	0.0579	0.7411
Print area LF	0.4734	**0.0054**	0.4338	**0.0073**	0.2116	0.2224
Print area LH	0.5766	**0.0004**	0.5278	**0.0008**	0.2436	0.1585
Print area RF	0.5098	**0.0024**	0.3924	**0.0163**	0.1445	0.4076
Print area RH	0.5870	**0.0003**	0.5633	**0.0003**	0.1218	0.4858
Maximum Intensity LF	0.6218	**0.0001**	0.3303	**0.0459**	0.1196	0.4938
Maximum Intensity LH	0.5538	**0.0008**	0.3638	**0.0269**	0.1374	0.4312
Maximum Intensity RF	0.6221	**0.0001**	0.5419	**0.0005**	0.1390	0.4258
Maximum Intensity RH	0.7365	**< 0.00001**	0.4507	**0.0051**	0.0827	0.6367
Base of support Front	0.5893	**0.0003**	0.2539	0.1294	0.1875	0.2808
Base of support Hind	0.3531	**0.0438**	− 0.0437	0.8005	0.5027	**0.0021**
Stride Length LF	0.0404	0.8234	− 0.1180	0.4867	0.2224	0.1991
Stride Length LH	− 0.1135	0.5312	0.0512	0.7635	0.2613	0.1295
Stride Length RF	0.0251	0.8897	− 0.1074	0.5285	0.1792	0.3030
Stride Length RH	− 0.0631	0.7276	− 0.0162	0.9251	0.2774	0.1067
Print position Left	0.3605	**0.0393**	0.0206	0.9037	− 0.2949	0.0865
Print position Right	0.4219	**0.0145**	0.0929	0.5845	− 0.1408	0.4225
Support Zero	0.1332	0.4599	− 0.0028	0.9906	0.1328	0.4470
Support Single	− 0.4605	**0.0071**	− 0.3671	**0.0255**	− 0.2217	0.2020
Support Diagonal	− 0.0210	0.9077	− 0.3175	0.0559	− 0.1558	0.3740
Support Girdle	− 0.0531	0.7696	0.0511	0.7639	− 0.0115	0.9500
Support Lateral	− 0.3794	**0.0296**	− 0.2155	0.2013	− 0.2132	0.2193
Support Three	0.3955	**0.0227**	0.3137	0.0587	0.2045	0.2386
Support Four	0.0126	0.9445	0.3272	**0.0481**	0.1333	0.4452
Phase dispersion LF-LH	− 0.0587	0.7485	0.0665	0.6958	0.0096	0.9564
Phase dispersion LF-RF	0.2387	0.1810	− 0.4299	**0.0081**	0.2815	0.1014
Phase dispersion LF-RH	0.0659	0.7156	0.1691	0.3171	− 0.0815	0.6437
Phase dispersion LH-RH	− 0.0710	0.6946	− 0.2202	0.1907	0.3408	**0.0451**
Phase dispersion RF-LH	0.0998	0.5805	− 0.0250	0.8832	− 0.4139	**0.0137**
Phase dispersion RF-RH	0.1737	0.3337	0.0547	0.7478	0.0116	0.9473
Number of patterns	− 0.3980	**0.0218**	0.1190	0.4830	− 0.2322	0.1799
Regularity Index	− 0.3763	**0.0310**	− 0.2003	0.2353	0.1721	0.3229
Step sequence-Aa	− 0.1789	0.3217	− 0.1426	0.4018	0.1733	0.3195
Step sequence-Ab	0.0893	0.6212	0.2169	0.1972	0.0541	0.7576
Step sequence-Ca	− 0.0726	0.6905	− 0.2661	0.1115	0.1350	0.4394
Step sequence-Cb	0.0372	0.8372	0.0886	0.6020	− 0.2398	0.1667
Step sequence-Ra	0.2701	0.1285	− 0.0839	0.6253	− 0.3772	**0.0256**
Step sequence-Rb	0.2180	0.2229	0.0813	0.6324	NA	NA

The correlation was measured using Pearson’s correlation coefficient for each strain. The bold values indicate significant results.

## Discussion

Gait analysis is a valuable and complementary state-of-the-art method for increasing the confidence in monitoring neuropsychiatric disorders-associated motor abnormalities. A normal gait depends on a complex interplay of major parts of the CNS, PNS, muscles, joints and cardiorespiratory system, and can indicate cognitive function, since intact cognition and attention processes are required to ensure proper gait performance^51,52^. Additionally, gait is a sensitive marker of the general health and survival of rodents^53^ and affects their behavioral outcome. Consequently, the study of gait performance should be performed as a part of the general phenotypic characterization of rodent models of neuropsychiatric disorders.

In our study, we aimed at evaluating the suitability and sensitivity of the CatWalk XT for gait characterization of three adolescent mouse strains, C57BL/6N, DBA/2, and FVB/N. To the best of our knowledge, the present study is the first to objectively and reliably quantify a large number of gait parameters of different mouse strains during adolescence. The same detection setting used for C57BL/6N and DBA/2 mice enabled a direct comparison between the two strains to provide a general idea about the difference in their gait performance during adolescence. A similar pattern of differences between the two strains was found at two ages during adolescence (P32 and P42) including the increased average, body and swing speed, enhanced spatial parameters, and decreased temporal ones in DBA/2 mice. Additionally, a similar pattern of differences in all interlimb coordination parameters except the number of patterns was found between the two strains at the two ages, suggesting a continuous strain effect at different ages during adolescence. Notably, the average speed of DBA/2 mice was higher than that of C57BL/6N mice at both ages. Since locomotor speed is known to affect several gait parameters in quadruples causing intraindividual and interindividual variability that are invisible in the statistical analysis^19,46–50,54–56^, the increased speed of DBA/2 mice can be a reason for the similar pattern difference at both ages. Indeed, the correlation analysis in adolescent mice revealed that around 60% of all gait parameters that we investigated (63 in total) are dependent on the locomotion speed of the mouse during the run. In a comprehensive study, the relationship between speed and 162 gait parameters in a group of 16 wild-type mice was investigated and revealed that over 90% of these parameters, reported by the CatWalk software, were dependent on speed^19^. Notably, that study investigated female B6SJLF1/J mice, generated by breeding C57BL/6J female with an SJL/J male, in comparison to our study that investigated both male and female mice of three different inbred strains. Our study was very similar to another study using male C57BL/6 mice revealing a negative correlation between speed and temporal parameters and a positive correlation with kinetic parameters such as swing and body speed with certain correlations to the spatial and interlimb coordination groups^46^. Although we showed a direct correlation of the speed with several gait parameters, we did not normalize our results comparing C57BL/6N and DBA/2 mice to speed since we were interested to examine the general gait performance of these strains including the effect of their speed as an intrinsic factor characterizing the strain. However, based on our study and the aforementioned ones, the speed of mice should be taken into consideration when characterizing the gait performance of rodents by a simple normalization of the speed or including it as a covariate during the statistical analysis of gait parameters. For mice modeling brain disorders that are expected to cause a shift in the gait performance irrelevant from their hyper/hypoactivity, it may be better to investigate the gait characteristics in a system that uses a controlled speed treadmill.

The high maximum intensity of all paws in DBA/2 mice at P32 indicates their increased propulsion, especially in hind paws^57^, which was absent at P42. Still, other factors may play a role in the similar increase in the maximum intensity of both front and hind paws. On the other hand, the increased print area of paws in DBA/2 mice may suggest a less efficient and incomplete clearance of the paw during locomotion compared to C57BL/6N mice, causing an increased hind paw contact with the floor rather than the normal contact of only the front part of the hind paw to the surface^58^. Since the CatWalk XT can not measure the absolute durations of paw clearance, the aforementioned assumption needs to be further validated using other gait analysis systems.

Interlimb coordination is a key characteristic of locomotion and, therefore, the parameters concerning coordination are of particular interest. There are six step-sequence patterns described in rodents, and the used pattern by rodents is known to differ because of either stress or a motor disease^56,58^. Our study revealed that the main step sequence pattern, Ab, was the same for the DBA/2 and FVB/N strains at both investigated ages. These results are in line with a previous study showing that intact rodents tend to prefer the Ab regular step pattern most of the time (80–95%)^59^. Interestingly, in our study, C57BL/6N mice showed a similar percentage of alternate and cruciate step sequences at P32 and P42. These results highlight the big effect of strain on interlimb coordination. For the support percentage, the longest simultaneous usage of paws during floor contact in all tested strains was with two diagonal paws, followed by three paws. These results indicate that interlimb coordination is a very stable gait characteristic that does not differ between mouse strains. This finding is in line with a previous study revealing no difference in interlimb coordination parameters in three rat strains, Sprague–Dawley, Wistar, and Lewis^55^. Notably, phase dispersions may be related to the support percentage since LF- > LH and RF- > RH dispersions are near 50%, especially in DBA/2 mice, matching the high percentage of their diagonal support.

The effect of sex on the body weights of mice was shown at P42 for C57BL/6N, DBA/2 and FVB/N mice. Regarding the effect of sex on gait performance, it was obvious mostly at P42 in the three investigated strains. Although these differences can be accounted by a change in the body weights of mice, especially the print area, base of support and regularity index that are shown to be correlated with body weight (Table 2), we believe that the differences in some gait parameters between males and females are dependent mainly on sex. Females are known to reach puberty before males, which can affect the gait performance during this sensitive period. However, the correlation between sex and body weight may still exist in some motor functions and should be kept into consideration when studying motor behaviors in rodents. Notably, the sex effect was dependent on the strain, with C57BL/6N showing sex differences in step cycle and base of support, DBA/2 showing sex differences in spatial parameters, and FVB/N showing sex differences in spatial parameters and base of support. Differences in the onset of puberty have been reported in different inbred strains^60^, which add another layer of complexity in comparing the behavioral results of different strains. Still, sex hormones and genes on X and Y chromosomes may have an impact on the behavioral outcome. In conclusion, our data confirm the strong intercorrelation between sex and strain on gait performance and highlight the importance of considering sex during gait analysis, even in young mice.

The 10-day comparison within the strain revealed differences on several parameters, indicating that gait performance is age-sensitive during adolescence. These differences were dependent on the investigated strain with C57BL/6N revealing differences on spatial parameters and base of support, DBA/2 showing differences on run characteristics, temporal parameters and base of support, and FVB/N showing differences mainly on swing time, spatial parameters, and stride length. These data confirm the intercorrelation between age and strain on gait performance.

Differences in body weights were found between the three mouse strains, which can be considered a possible confounder of their gait data^47,55^. Indeed, the correlations between the body weights of mice and CatWalk XT gait parameters were found mainly in the spatial parameters of both C57BL/6N and DBA/2 but not FVB/2 mice. The increase in body weights causing increased print areas and maximum intensities of paws and increasing the percentage of support on three and four paws instead of a single paw can have a direct effect on the gait performance and the number of gait patterns. These results indicate the importance of adding the weights of mice into consideration when evaluating the gait performance and motor function, as previously shown^41^. Additionally, since the body weights of FVB/N mice did not show similar correlations to spatial parameters like C57BL/6N and DBA/2 mice, this highlights the intercorrelation between strain and body weight. Still, we believe that the strain has a stronger impact than the body weights of mice on their gait performance. This hypothesis is confirmed by our results showing that DBA/2 mice at P42 had increased print areas of paws despite their decreased body weights compared to C57BL/6N mice.

The correlation between body weight and gait performance has been previously investigated in several studies in adult rodents. In two studies in rodents with middle cerebral artery occlusion (MCAO), body weight showed a strong correlation with several parameters including stand, step cycle, and print area of paws^46,50^. In contrast, two other studies have shown no correlation between body weight and any of the measured CatWalk XT gait parameters in mouse and rat models of stroke^57,61^. The inconsistent results between different studies can be partially explained by the different species, strains, models of diseases, and statistical tests used (Pearson’s vs Spearman’s correlation coefficients; *p* value ≤ 0.05 vs adjusted *p* value for correction of multiple comparisons). These variabilities in different studies need to be reduced or, at least, taken into consideration for achieving reproducible results between laboratories.

In our previous study, we compared the motor and coordination functions of these three inbred strains in six behavioral tests: grip strength, beam balance rod, inverted screen, cliff avoidance reaction, rotarod, and voluntary wheel running^41^. The results of that study showed that most of these experiments can be performed reliably on young mice with DBA/2 mice having less motor and coordination abilities^41^. How these results can be correlated with gait performance needs to be further investigated. The increased average, body and swing speeds of DBA/2 mice may have a negative effect on coordination. Additionally, the increased print areas of all paws can be a sign of incomplete clearance of paws during locomotion, which decreases the efficiency of the whole process. This hypothesis can be strengthened by the increased print areas of the right paws in male DBA/2 mice compared to females at P42, which is consistent with the better performance of female mice on the rotarod^41^. Still, the reduced body weight of DBA/2 mice can result in a decrease in the muscle strength as shown previously in the grip strength test^41^ and may have an impact on the motor ability in the rotarod that requires high endurance.

Other devices including DigiGait (Mouse Specifics Inc., Framingham, MA), TreadScan (CleverSys Inc., Reston, VA), and Dynamic Weight Bearing test are well known to study the gait of rodents^13,62,63^. DigiGait and TreadScan have similar equipment, including a treadmill driving rodents into passive walking/running, which requires extra balance, and thus shorter strides and increased stance times^62^. In contrast, the CatWalk system uses a stable track that enables rodents to actively move forward. Differences in results using different devices are reported^62,64^, which can be explained by differences in the relevant task (passive vs active movements), equipment size, color, and lighting. This highlights that even small methodological factors play an important role in the gait analysis of rodents. Recently, new high-level software with an intuitive, easy-to-use interface built on DeepLabCut™ was used for the analysis of gait performance of mice and named Visual Gait Lab (VGL)^65^. The main advantage of VGL over the CatWalk system is the visual tracking of absolute positions of the body and the paws, rather than differentiating paw prints compared to back-ground intensity, which can be unreliable with injured mouse models where paw contact with the ground is compromised^66^. Therefore, it will be of interest to validate the results of our study using another gait analysis system.

There are some limitations of the CatWalk system including the high dependence on the rodent’s behavior during examination (i.e. constant locomotor speed, trajectory) and their motivation to complete the task. These limitations can be aggravated by using adolescent mice that are known to show an intrinsic higher activity, to be more anxious, sensitive to social isolation, and have a lower capability to learn the task. Therefore, data acquisition of gait performance can be affected due to interrupted walk and heterogeneous velocity, demanding for taking care of the data interpretation. Another limitation of the CatWalk system is the difficulty to make a complete interpretation of all gait parameters, especially when they are evaluated individually. For unraveling the effect of neuropsychiatric disorders on motor functions, understanding the definition of every gait parameter is required, and the reason for each change needs a comprehensive determination and consideration of how multiple different parameters contribute to an altered gait pattern. Indeed, simple combinations of CatWalk parameters have been employed in a variety of disorders to assess gait, such as baseline parameter ratio, left–right-parameter ratio, left–right-parameter averaging, and subtraction^67–73^. Recently, Timotius et al. developed a CatWalk gait parameter combination for sensitive and efficient gait recovery assessment in a rat thoracic spinal cord injury model by combining nine of the topmost parameters into a gait index score based on linear discriminant analysis^74^. The analysis of different combinations of gait parameters more efficiently represents the fully-coordinated motion of gait than single parameters and enhances the evaluation power of gait performance in rodent models of neuropsychiatric disorders.

## Conclusion

Our results can serve as a template for studying changes in locomotor and gait performance in C57BL/6N, DBA/2, and FVB/N adolescent mice. Moreover, the present report confirmed that genetic strain differences are important determinants of motor and gait functions. Having unraveled age-, sex-, speed- and weight-dependence of gait performance, we recommend littermates of the same delivery day, and alike male/female ratios for the behavioral characterization of knockout/knockin mice, as well as correcting for the speed and body weight during the statistical analysis. Keeping these factors in mind will allow a reliable characterization of the motor activity and coordination functions in rodent models of neuropsychiatric disorders.

## Supplementary Information

Supplementary Information.
