# Supplementary material for: Gait performance of adolescent mice assessed by the CatWalk XT depends on age, strain and sex and correlates with speed and body weight
Source: Sci Rep. 2021 Nov 1;11:21372. doi: 10.1038/s41598-021-00625-8 (PMC8560926; doi:10.1038/s41598-021-00625-8)
Supplement: Supplementary file 1 — Supplementary Information. [file 41598_2021_625_MOESM1_ESM.docx]

**Gait performance of adolescent mice assessed by the CatWalk XT depends on age, strain and sex and correlates with speed and body weight**

Claudia Pitzer^1^*, Barbara Kurpiers^1^, Ahmed Eltokhi^2^*

^1^ Interdisciplinary Neurobehavioral Core, Heidelberg University, Heidelberg, Germany

^2^ Department of Pharmacology, University of Washington, Seattle, USA

*Correspondence: Dr. Claudia Pitzer: Claudia.pitzer@pharma.uni-heidelberg.de, Dr. Ahmed Eltokhi: Eltokhi@uw.edu


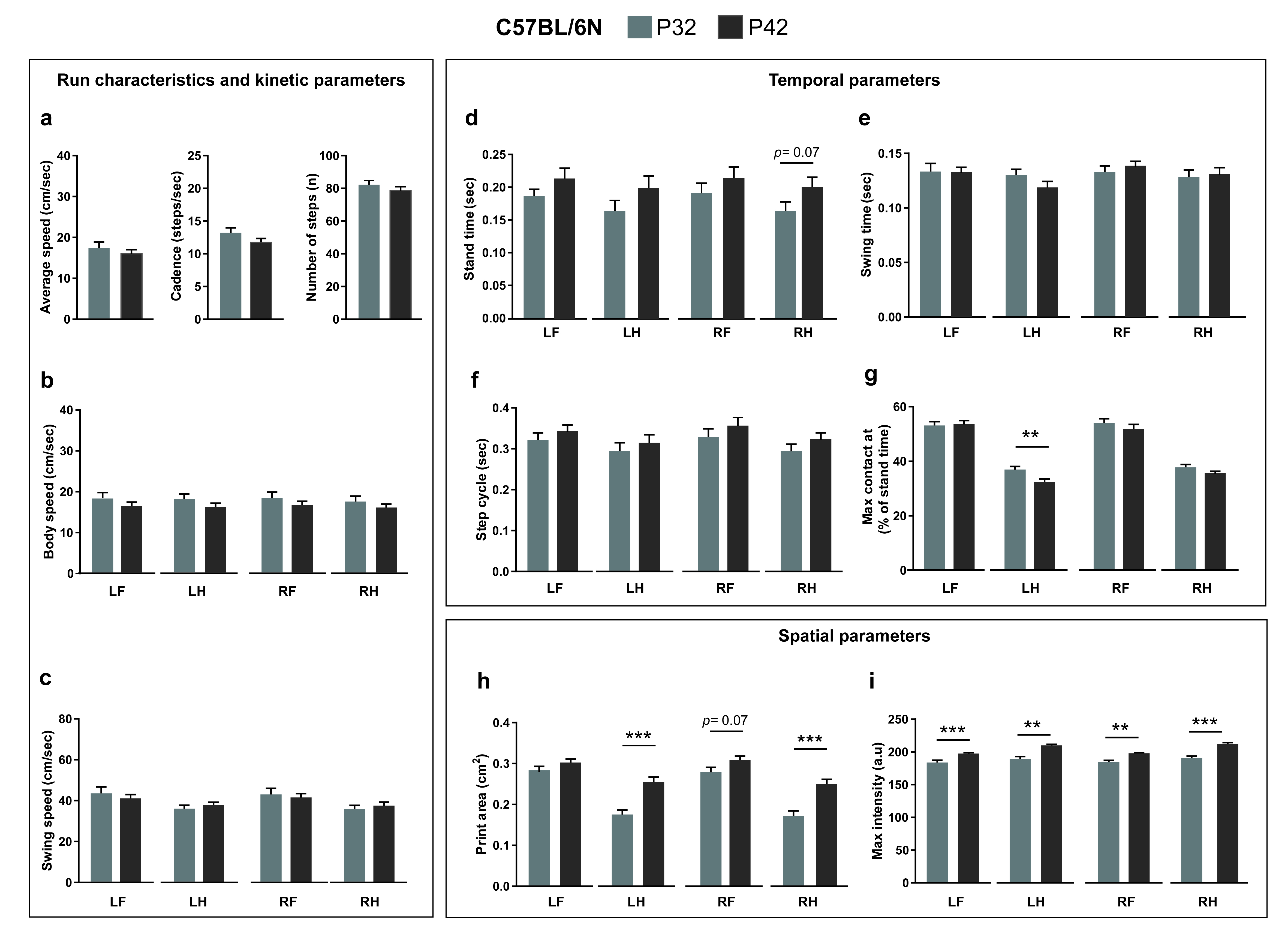


**Supplementary Figure 1: Comparison of run characteristics and kinetic, temporal and spatial parameters between P32 and P42 C57BL/6N mice**

**(a-c)** No difference in the average speed, cadence, number of steps **(a)**, body speed **(b)** or swing speed **(c)** between P32 and P42 C57BL/6N mice. **(d-f)** No significant difference in the stand time **(d),** swing time **(e)** or step cycle **(f)** between P32 and P42 C57BL/6N mice. **(g)** The maximum contact at (% of stand time) of the LH paw was significantly less at P42 than at P32. **(h)** The print areas of both LH and RH paws were significantly increased at P42 compared to at P32. **(i)** The maximum intensities of all paws at P42 were more than at P32. Two-way ANOVA followed by Bonferroni post hoc test, ***p* ≤ 0.01, ****p* ≤ 0.001. Error bars indicate the standard error of the mean (SEM). RF: right front; LF: left front; RH: right hind; LH: left hind.


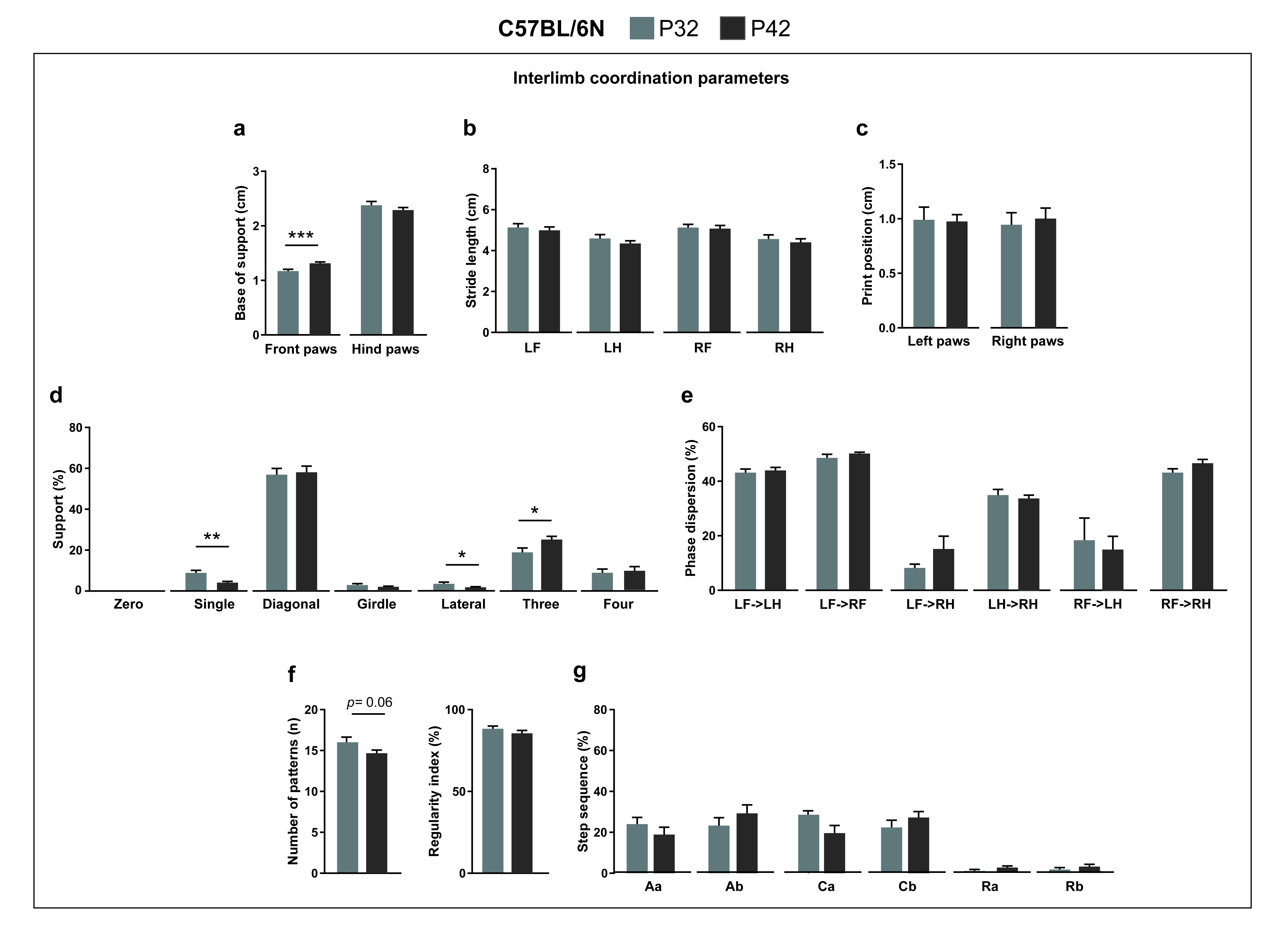


**Supplementary Figure 2: Comparison of interlimb coordination parameters between P32 and P42 C57BL/6N mice**

The base of support of the front paws was higher at P42 than at P32. **(b-c)** No difference in the stride lengths (b) or print positions (c) of all paws between P32 and P42. **(d)** The percentages of support on single and lateral paws were significantly less at P42 than at P32. In contrast, the percentage of support on three paws was increased at P42. **(e-g)** No difference in the phase dispersion **(e)**, number of patterns, regularity index **(f)** or step sequence **(g)** between P32 and P42 C57BL/6N mice. Two-way ANOVA followed by Bonferroni post hoc test, **p* ≤ 0.05, ***p* ≤ 0.01, ****p* ≤ 0.001. Error bars indicate the standard error of the mean (SEM). RF: right front; LF: left front; RH: right hind; LH: left hind.


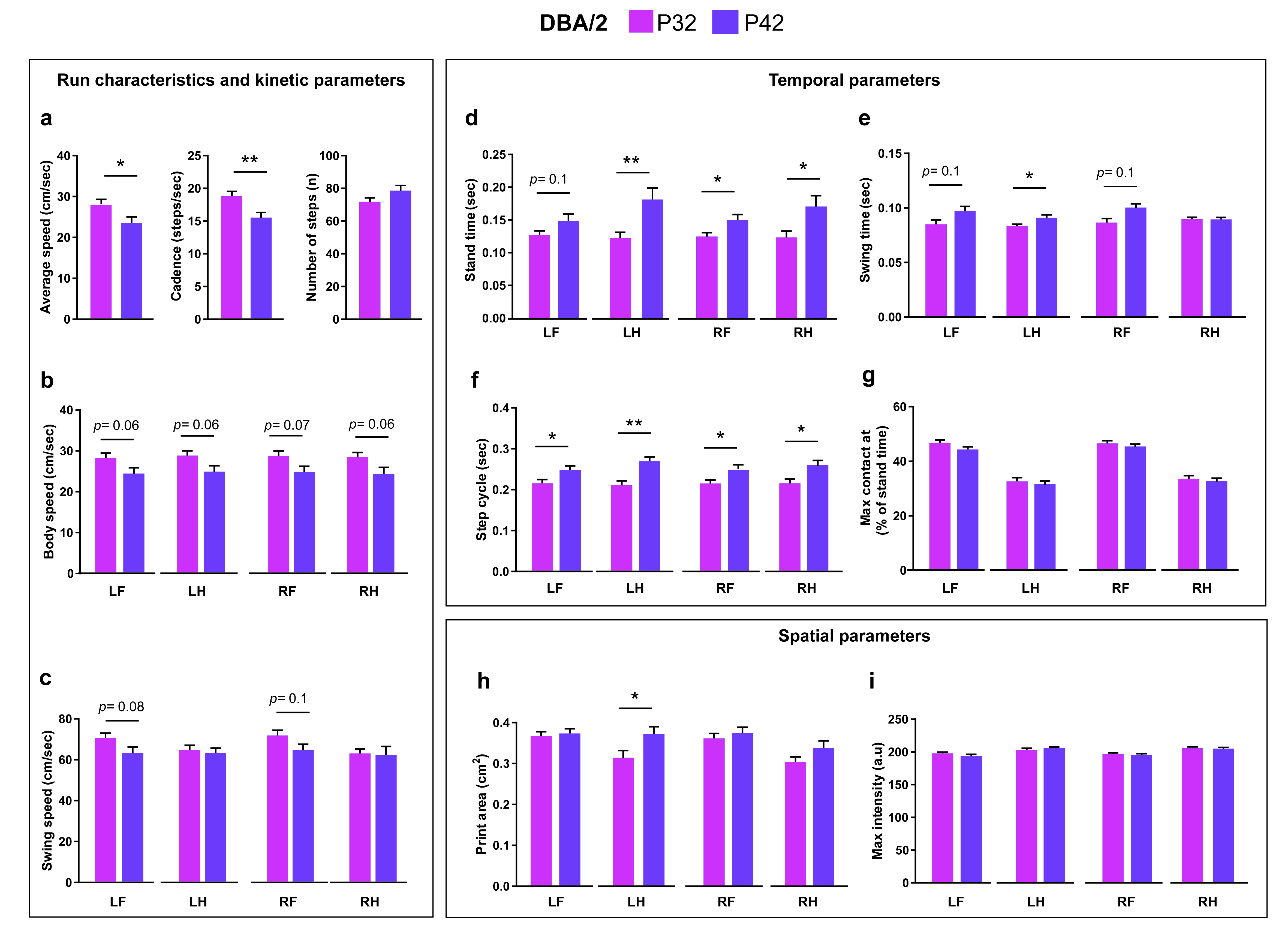


**Supplementary Figure 3: Comparison of run characteristics and kinetic, temporal and spatial parameters between P32 and P42 DBA/2 mice**

**(a)** P42 DBA/2 mice showed less average speed and cadence compared to P32 mice. No difference in the number of steps was found. **(b)** P42 DBA/2 mice showed a borderline significant less body speed of all paws compared to P32 mice. **(c)** No difference in the swing speed was found between P32 and P42 DBA/2 mice. **(d)** The stand times of LH, RF and RH paws were significantly higher at P42 than at P32. **(e)** The swing times of hind paws were increased at P42 compared to P32. **(f)** The step cycles of all paws at P42 were significantly more than at P32. **(g)** No difference between the max contact at (% of stand time) of all paws between P32 and P42 DBA/2 mice. **(h)** The print area of LH paw was more at P42 than P32. **(i)** No difference in the maximum intensity of all paws was found between P32 and P42. Two-way ANOVA followed by Bonferroni post hoc test, **p* ≤ 0.05, ***p* ≤ 0.01, ****p* ≤ 0.001. Error bars indicate the standard error of the mean (SEM). RF: right front; LF: left front; RH: right hind; LH: left hind.


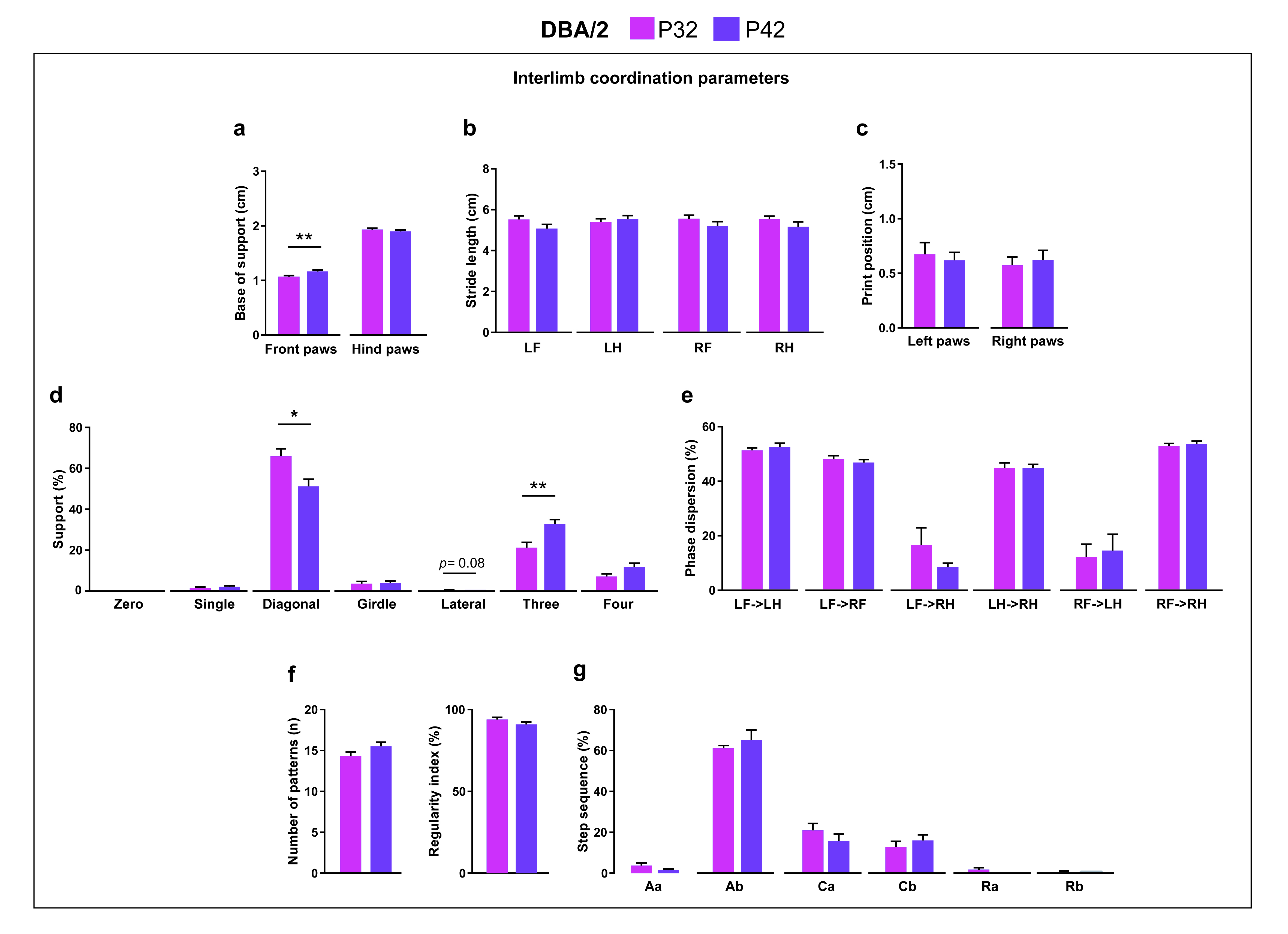


**Supplementary Figure 4: Comparison of interlimb coordination parameters between P32 and P42 DBA/2 mice**

**(a)** The base of support of front paws was higher at P42 than P32. **(b-c)** No difference in the stride lengths **(b)** or print positions **(c)** of all paws between P32 and P42. **(d)** The percentage of support on diagonal paws was significantly less at P42 than at P32. In contrast, the percentage of support on three paws was increased at P42. **(e-g)** No difference in the phase dispersion **(e)**, number of patterns, regularity index **(f)** or step sequence **(g)** between P32 and P42 FVB/N mice. Two-way ANOVA followed by Bonferroni post hoc test, **p* ≤ 0.05, ***p* ≤ 0.01. Error bars indicate the standard error of the mean (SEM). RF: right front; LF: left front; RH: right hind; LH: left hind.

**Supplementary Table 1: Gait parameters showing significant differences between male and female mice within at least one strain.** The yellow highlights indicate significant results.

|  | **C57BL/6N** | | | **DBA/2** | | | **FVB/N** | | |
| --- | --- | --- | --- | --- | --- | --- | --- | --- | --- |
| **Parameters** | ♂ Mean | ♀ Mean | Bonferroni test | ♂Mean | ♀ Mean | Bonferroni test | ♂Mean | ♀ Mean | Bonferroni test |
| **P32** | | | | | | | | | |
| **Temporal parameters** | | | | | | | | | |
| Step cycle (RF) | 0.37 | 0.30 | 0.0457 | 0.21 | 0.23 | 0.6949 | 0.25 | 0.24 | 0.9942 |
| **Interlimb coordination parameters** | | | | | | | | | |
| Base of support (Hind Paws) | 2.29 | 2.44 | 0.2983 | 1.95 | 1.93 | >0.9999 | 1.84 | 1.64 | 0.0026 |
| Support (single) | 6.35 | 10.56 | 0.0411 | 1.30 | 2.11 | >0.9999 | 0.53 | 1.88 | 0.5143 |
| Support (Girdle) | 3.79 | 2.39 | >0.9999 | 1.79 | 6.16 | 0.0371 | 1.00 | 0.51 | 0.5819 |
| Support (Lateral) | 2.82 | 3.85 | 0.6712 | 0.48 | 0.54 | >0.9999 | 1.37 | 3.65 | 0.0189 |
|  |  |  |  |  |  |  |  |  |  |
| **P42** | | | | | | | | | |
| Body weight at P42 | 19.40 | 16.67 | <0.0001 | 18.39 | 15.60 | 0.0001 | 21.79 | 19.17 | <0.0001 |
| **Kinetic parameters** | | | | | | | | | |
| Swing Speed (RH) | 35.26 | 39.71 | 0.9167 | 65.19 | 59.09 | 0.6806 | 76.89 | 63.81 | 0.0317 |
| **Temporal parameters** | | | | | | | | | |
| Stand time (LF) | 0.26 | 0.18 | 0.0041 | 0.16 | 0.14 | >0.9999 | 0.14 | 0.14 | >0.9999 |
| Stand time (RF) | 0.26 | 0.18 | 0.0053 | 0.16 | 0.14 | 0.6653 | 0.14 | 0.14 | >0.9999 |
| Step cycle (LF) | 0.39 | 0.30 | 0.0072 | 0.26 | 0.24 | 0.7638 | 0.25 | 0.26 | >0.9999 |
| Step cycle (LH) | 0.37 | 0.27 | 0.0309 | 0.28 | 0.26 | >0.9999 | 0.25 | 0.25 | >0.9999 |
| Step cycle (RF) | 0.41 | 0.32 | 0.0076 | 0.26 | 0.25 | >0.9999 | 0.25 | 0.26 | 0.6387 |
| **Spatial parameters** | | | | | | | | | |
| Print area (LF) | 0.33 | 0.28 | 0.0598 | 0.38 | 0.35 | 0.2819 | 0.35 | 0.27 | 0.0336 |
| Print area (RF) | 0.32 | 0.30 | 0.6412 | 0.40 | 0.34 | 0.0280 | 0.34 | 0.26 | 0.0293 |
| Print area (RH) | 0.27 | 0.23 | 0.2891 | 0.38 | 0.29 | 0.0034 | 0.26 | 0.21 | 0.1192 |
| Maximum intensity (RF) | 197.20 | 197.20 | >0.9999 | 199.70 | 189.60 | 0.0051 | 124.30 | 112.30 | 0.0472 |
| **Interlimb coordination parameters** | | | | | | | | | |
| Base of support (Hind paws) | 2.45 | 2.17 | <0.0001 | 1.96 | 1.84 | 0.1402 | 1.84 | 1.56 | <0.0001 |
| Support (Diagonal) | 50.29 | 64.36 | 0.0487 | 47.51 | 54.65 | 0.5324 | 67.31 | 69.85 | >0.9999 |
| Phase dispersion (LF->RF) | 50.36 | 49.76 | >0.9999 | 45.27 | 49.49 | 0.0219 | 49.70 | 48.73 | >0.9999 |
| Phase dispersion (RF->LH) | 20.47 | 11.01 | 0.7539 | 19.1 | 9.46 | 0.7929 | 5.49 | 49.70 | 0.0091 |
| Regularity index | 80.71 | 89.47 | 0.0077 | 90.56 | 91.48 | >0.9999 | 96.31 | 94.18 | 0.4225 |
